# MicroRNA-30 regulates left ventricular hypertrophy in chronic kidney disease

**DOI:** 10.1172/jci.insight.138027

**Published:** 2021-05-24

**Authors:** Jingfu Bao, Yinghui Lu, Qinying She, Weijuan Dou, Rong Tang, Xiaodong Xu, Mingchao Zhang, Ling Zhu, Qing Zhou, Hui Li, Guohua Zhou, Zhongzhou Yang, Shaolin Shi, Zhihong Liu, Chunxia Zheng

**Affiliations:** 1 National Clinical Research Center of Kidney Diseases, and; 2 Department of Pharmacology, Jinling Hospital, Nanjing University School of Medicine, Nanjing, China.; 3 State Key Laboratory of Pharmaceutical Biotechnology, Department of Cardiology, Nanjing Drum Tower Hospital, The Affiliated Hospital of Nanjing University School of Medicine, and MOE Key Laboratory of Model Animal for Disease Study, Model Animal Research Center, Nanjing University, Nanjing, China.

**Keywords:** Cardiology, Nephrology, Cardiovascular disease, Chronic kidney disease

## Abstract

Left ventricular hypertrophy (LVH) is a primary feature of cardiovascular complications in patients with chronic kidney disease (CKD). miRNA-30 is an important posttranscriptional regulator of LVH, but it is unknown whether miRNA-30 participates in the process of CKD-induced LVH. In the present study, we found that CKD not only resulted in LVH but also suppressed miRNA-30 expression in the myocardium. Rescue of cardiomyocyte-specific miRNA-30 attenuated LVH in CKD rats without altering CKD progression. Importantly, in vivo and in vitro knockdown of miRNA-30 in cardiomyocytes led to cardiomyocyte hypertrophy by upregulating the calcineurin signaling directly. Furthermore, CKD-related detrimental factors, such as fibroblast growth factor-23, uremic toxin, angiotensin II, and transforming growth factor–β, suppressed cardiac miRNA-30 expression, while miRNA-30 supplementation blunted cardiomyocyte hypertrophy induced by such factors. These results uncover a potentially novel mechanism of CKD-induced LVH and provide a potential therapeutic target for CKD patients with LVH.

## Abstract 2

Downregulation of myocardial miRNA-30 is involved in chronic kidney disease–induced left ventricular hypertrophy, whereas exogenous miRNA-30 rescue inhibits this process.

## Graphical abstract

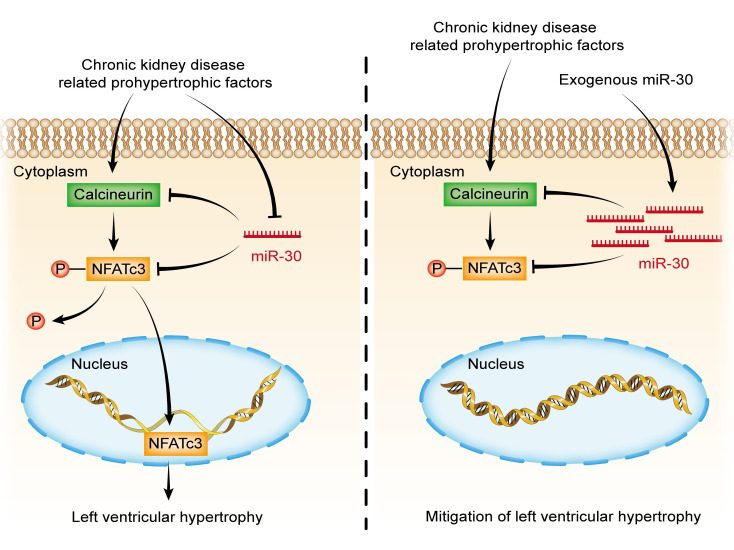

## Introduction

Cardiovascular complications are now considered a major factor that affects the prognosis of patients with chronic kidney disease (CKD) (1, 2). Increased knowledge about the epidemiology of CKD-related cardiovascular disease gives us insight into the leading cause of death for patients with CKD (3–6). Left ventricular hypertrophy (LVH) is a common feature of cardiac changes in CKD and contributes to more severe cardiovascular anomalies (7). LVH presents even in very early CKD stages and occurs in up to 65% of predialysis patients (8). Hemodynamic overload is a well-known inducer of LVH development in patients with CKD (9), whereas other CKD-related detrimental factors, such as the renin-angiotensin system (RAS), uremic toxin, microinflammatory state, and phosphorus metabolism disorder, are all closely associated with LVH (10). Identification of a common mechanism in cardiac hypertrophy induced by such pathogenic factors could provide an understanding of the CKD-induced LVH, leading to more reliable therapy for CKD patients with cardiovascular complications.

miRNAs are a class of short noncoding RNAs that regulate gene expression at the posttranscriptional level (11), and they can be found in a large number of biological processes, including cardiac hypertrophy (12). Numerous miRNAs with important roles in cardiac hypertrophy have been identified, such as miRNA-1 (13–15), miRNA-9 (16), and miRNA-133 (17–20). The miRNA-30 family (miR-30) is highly expressed in the heart (21–23) and is closely related to cardiac remodeling (24). It has been reported that miR-30 is downregulated in the heart from cardiac hypertrophy or in heart failure models (25), and such an expression pattern can also be found in diabetic cardiomyopathy (26). Mechanistically, miR-30 can regulate autophagy (27–30), apoptosis (26, 30–34), and oxidative stress (30, 32), which are all related to cardiac hypertrophy. Hence, we hypothesized that disturbed expression of myocardial miR-30 may act as a common mediator of LVH progression in CKD.

Here, we performed a series of experiments to confirm the role of miR-30 in the development of CKD-induced LVH. We demonstrated that subtotal nephrectomy (SN) is sufficient to induce LVH and miR-30 suppression in the heart. Cardiac miR-30 rescue inhibited the progression of LVH in these CKD models. Mechanistic experiments further revealed that calcineurin signaling associated miR-30 with LVH. In addition, we found that prohypertrophic stimuli were inducers of cardiac miR-30 suppression, whereas their prohypertrophic effects could be blocked by miR-30. These results support a model to illustrate the pivotal role of miR-30 in CKD-induced LVH and provide evidence for the potential therapeutic role of miR-30 in CKD-related cardiovascular complications.

## Results

### Time-dependent effects of SN on the heart.

**Figure 1:**
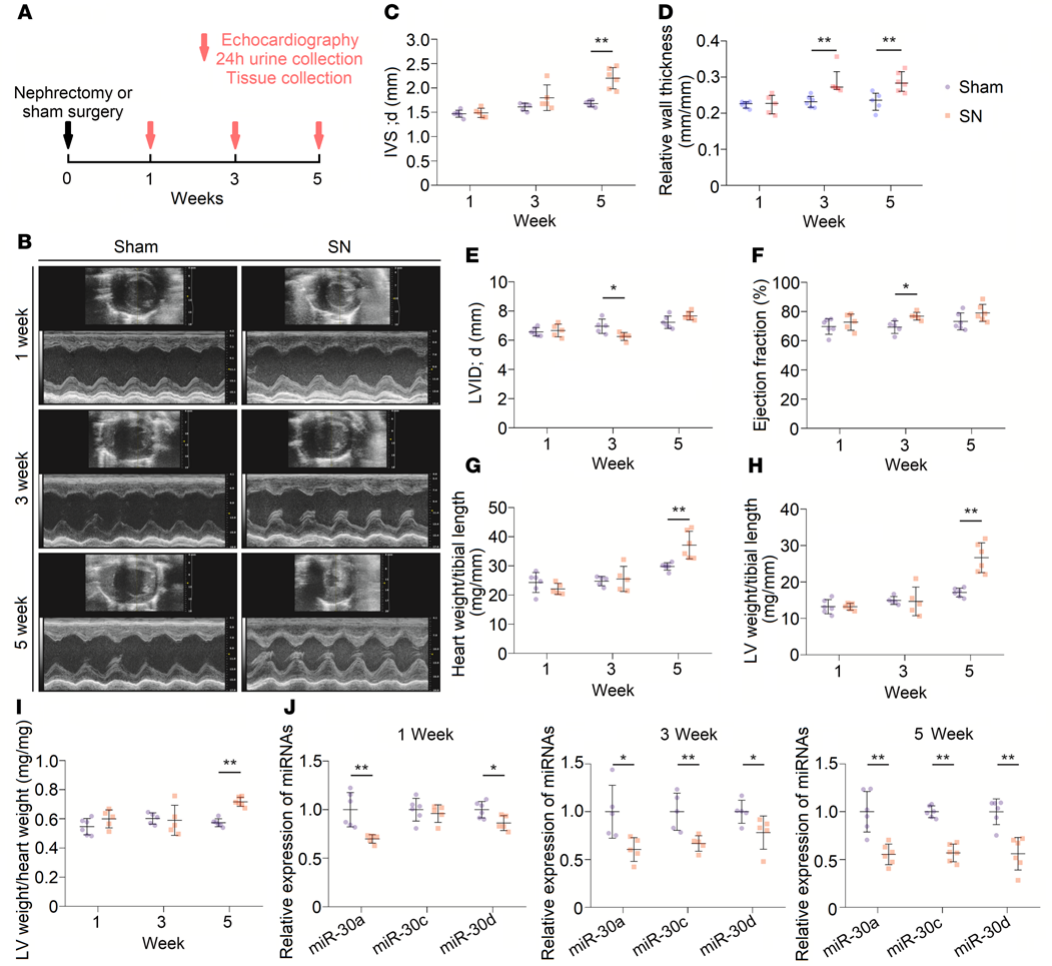
LVH gradually appears in SN rats. (**A**) Schematic diagram for continuous observation in nephrectomized rats. (**B**) Representative short-axis echocardiography and M-mode images. (**C** and **D**) Diastolic interventricular septal thickness (IVS; d) and relative wall thickness of left ventricles gradually increase in nephrectomized rats. **P < 0.01 compared with values for the sham indicated by the dashed line, by 2-tailed, unpaired Student’s *t* test (**C**) and by Mann-Whitney *U* test (**D**). Data are shown as mean ± SD and median with interquartile range (**C** and **D**, respectively). *n* = 5 or 6 rats per group. (**E**) Diastolic LV internal diameter (LVID; d) decreases at 3 weeks after nephrectomy. *P < 0.05 compared with values for the sham indicated by the dashed line, by 2-tailed, unpaired Student’s *t* test. Data are shown as mean ± SD. *n* = 5 or 6 rats per group. (**F**) Nephrectomy leads to LV ejection fraction increase at 3 weeks after nephrectomy. *P < 0.05 compared with values for the sham indicated by the dashed line, by 2-tailed, unpaired Student’s *t* test. Data are shown as mean ± SD. *n* = 5 or 6 rats per group. (**G**–**I**) SN results in increase in heart weight/tibial length, LV weight/tibial length, and LV weight/heart weight ratios at 5 weeks after surgery. **P < 0.01 compared with values for the sham indicated by the dashed line, by 2-tailed, unpaired Student’s *t* test. Data are shown as mean ± SD. *n* = 5 or 6 rats per group. (**J**) Nephrectomy leads to downregulation of cardiac miR-30. Expression levels are normalized by *U6*. *P < 0.05 and **P < 0.01 compared with values for the sham indicated by the dashed line, by 2-tailed, unpaired Student’s *t* test. Data are shown as mean ± SD. *n* = 5 or 6 rats per group.

To construct an appropriate CKD-induced LVH model, we performed SN in male Sprague-Dawley rats (35). The body weights of nephrectomized rats were reduced at 1 week after surgery, and delayed body growth became increasingly obvious (Supplemental Figure 1A; supplemental material available online with this article; https://doi.org/10.1172/jci.insight.138027DS1). Renal system–related impairments were continuously observed via biochemical values of serum and kidney tissue slices at 1, 3, and 5 weeks after surgery (Figure 1A). Renal insufficiencies were apparent in SN rats, as indicated by increased serum creatinine and serum urea nitrogen and a decreased creatinine clearance rate (Supplemental Figure 1, B–D). The average systolic blood pressure and diastolic blood pressure showed a gradually increasing trend in nephrectomized rats as well (Supplemental Figure 1, E and F). Remnant kidney tissues from nephrectomized rats presented trends of tubular dilation and interstitial fibrosis (Supplemental Figure 1G).

Sequential cardiac changes were analyzed by echocardiography at 1, 3, and 5 weeks after surgery (Figure 1A). Echocardiographic analysis showed progressive thickening of the left ventricular (LV) wall (Figure 1, C and D), and a relative reduction of chamber diameter was observed at 3 weeks after surgery in SN rats (Figure 1E). Representative short-axis echocardiography and M-mode images are shown in Figure 1B. Interestingly, nephrectomized rats exhibited a relatively higher ejection fraction of the left ventricle, although statistical significance was only observed at 3 weeks after surgery (Figure 1F). Detailed measurements of short-axis echocardiography are shown in Table 1. Moreover, there were significant increases in heart weight/tibial length, LV weight/tibial length, and LV weight/heart weight ratios at 5 weeks after surgery (Figure 1, G–I). Collectively, these results indicated that a CKD-induced LVH model was successfully established in Sprague-Dawley rats.

### Cardiac miR-30 is downregulated in CKD.

A previous study showed that miR-30a, miR-30c, and miR-30d are the 3 most highly expressed types of miR-30 in mouse (21) and rat (22) myocardial tissues; hence, we performed TaqMan quantitative polymerase chain reaction (qPCR) to detect the alterations in these 3 members of miR-30 in cardiac tissues from the abovementioned rat model. We found that myocardial miR-30a, miR-30c, and miR-30d were downregulated, and the longer the kidney injury lasted, the lower the cardiac miR-30 observed (Figure 1J). In addition, we investigated the cardiac expression of miR-30e at 5 weeks after surgery because it is expressed at the same levels as miR-30c in rat cardiac tissue (22). Consistently, we observed reduced cardiac expression of miR-30e in CKD rats (Supplemental Figure 2). These results suggested a downregulated expression pattern of myocardial miR-30 in CKD.

### Cardiac miR-30 rescue mitigates LVH in CKD.

**Figure 2:**
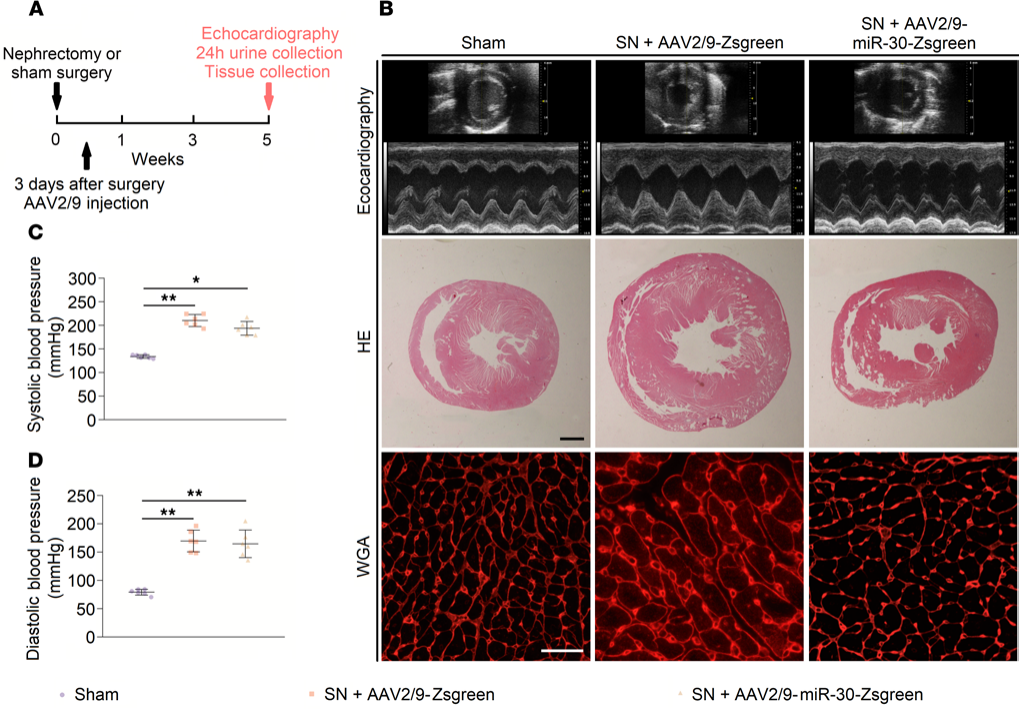
AAV2/9 injection in sham and nephrectomized rats. (**A**) Schematic diagram for AAV2/9 injection and efficacy observation. (**B**) Representative short-axis echocardiography and M-mode images, cross sections (hematoxylin and eosin staining; original magnification, ×6.3; scale bar: 1000 μm), and WGA staining (original magnification, ×400; scale bar: 50 nm) of myocardium. (**C** and **D**) Cardiac miR-30 rescue does not affect blood pressures in SN rats. *P < 0.05, **P < 0.01 compared with values indicated by the dashed line, by 1-way ANOVA test. Tukey’s multiple comparisons test was used for multiple comparison. Data are shown as mean ± SD. *n* = 6 rats per group.

Since CKD-induced LVH is accompanied by downregulated cardiac miR-30, we speculated that miR-30 can exert antihypertrophic effects. We chose adeno-associated virus 2 serotype 9 (AAV2/9), which has a high affinity for cardiomyocytes (36), to deliver the miR-30 expression plasmid into the cardiomyocytes of CKD rats. Our previous study constructed an expressed miR-30a-miR-30c-miR-30d-sequence, which can recover miR-30 functions in injured podocytes (37). Thus, we loaded this expressed sequence into AAV2/9, and its expression was controlled by the cardiac troponin T (cTnT) promoter. Following miR-30 sequence, a cytomegalovirus enhancer– and promoter-driven *Zoanthus sp.* green florescent protein (Zsgreen) sequence was loaded into AAV2/9. Two types of viruses were generated, namely AAV2/9-miR-30-Zsgreen and AAV2/9-Zsgreen (Supplemental Figure 3). Between these 2 types, AAV2/9-Zsgreen was used as the blank control. Subsequently, we performed SN in male Sprague-Dawley rats and delivered either 10^11^ viral genome copies (vg) AAV2/9-miR-30-Zsgreen or 10^11^ vg AAV2/9-Zsgreen by subclavian vein injection to each nephrectomized rat 3 days after surgery (Figure 2A). We measured the blood pressures, performed echocardiography, and collected blood and tissues 5 weeks after surgery. Confocal microscopy showed Zsgreen expression in cardiomyocytes that received AAV2/9 injection (Supplemental Figure 4A). TaqMan qPCR results indicated that myocardial miR-30 was effectively supplemented by AAV2/9 transfection (Supplemental Figure 4B).

Compared with those of the sham group, the body growth and renal function of nephrectomized rats were significantly impaired. The body weights of nephrectomized rats were significantly reduced, and AAV2/9 injection did not inhibit this decrease (Supplemental Figure 5A). Moreover, the SN groups showed increased serum creatinine and urea nitrogen with a decreased creatinine clearance rate, but there was no difference between the SN groups that received AAV2/9-miR-30-Zsgreen or AAV2/9-Zsgreen (Supplemental Figure 5, B–D). Histology of remnant kidney tissues revealed similar changes in nephrectomized rats that received AAV2/9-miR-30-Zsgreen or AAV2/9-Zsgreen (Supplemental Figure 5E).

**Figure 3:**
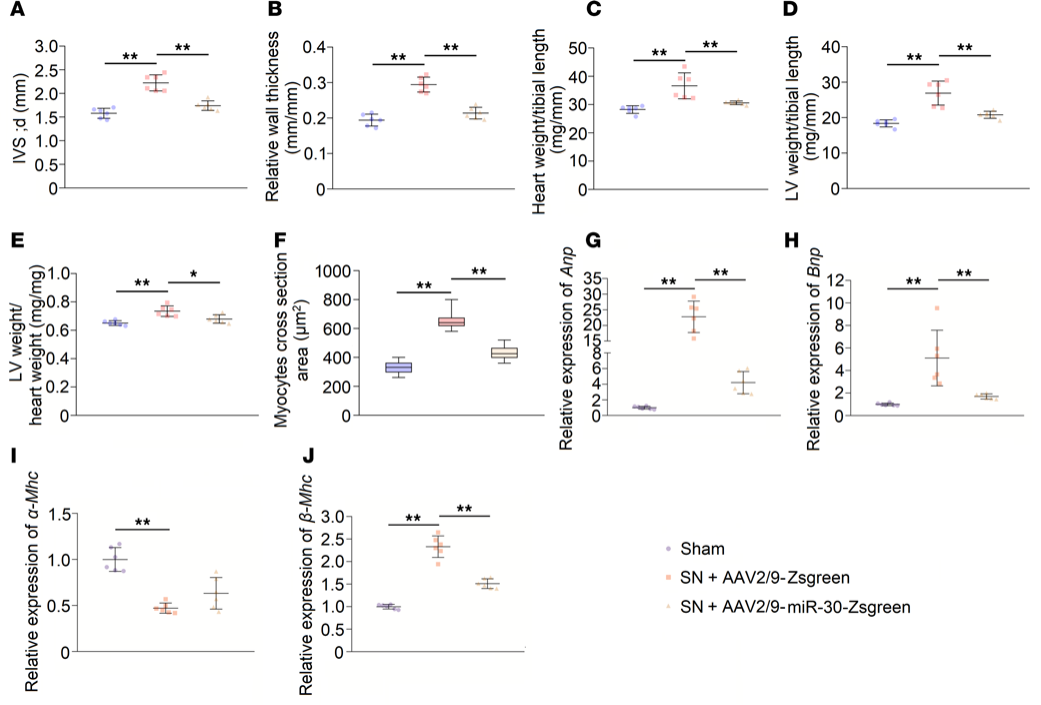
Cardiac miR-30 rescue mitigates CKD-induced LVH. (**A** and **B**) miR-30 inhibits the IVS; d and relative wall thickness of LV increases in nephrectomized rats. **P < 0.01 compared with values indicated by the dashed line, by 1-way ANOVA test. Tukey’s multiple comparisons test was used for multiple comparison. Data are shown as mean ± SD. *n* = 6 rats per group. (**C**–**E**) miR-30 decreases heart weight/tibial length, LV weight/tibial length, and LV weight/heart weight ratios in SN rats. *P < 0.05 and **P < 0.01 compared with values indicated by the dashed line, by 1-way ANOVA test. Tukey’s multiple comparisons test was used for multiple comparison. Data are shown as mean ± SD. *n* = 6 rats per group. (**F**) MiR-30 suppresses increased cross-sectional area of cardiomyocytes in nephrectomized rats. **P < 0.01 compared with values indicated by the dashed line, by 1-way ANOVA test. Tukey’s multiple comparisons test was used for multiple comparison. Data are shown as median and quartiles, as well as the minimum and maximum values of the distribution. *n* = 360 cells per group. (**G**–**J**) miR-30 mitigates upregulated hypertrophic indicators in CKD rats, despite having no significant effect on α–myosin heavy chain (*α-Mhc*). Expression levels are normalized by *18S*. **P < 0.01 compared with values indicated by the dashed line, by 1-way ANOVA test. Tukey’s multiple comparisons test was used for multiple comparison. Data are shown as mean ± SD. *n* = 6 rats per group.

Regarding cardiac changes, SN rats that were injected with AAV2/9-Zsgreen developed cardiac hypertrophy, whereas AAV2/9-miR-30-Zsgreen-treated SN rats showed decreased ventricular wall thickness and relative wall thickness (Figure 3, A and B). Of note, AAV2/9 injection did not affect the increased blood pressure in nephrectomized rats (Figure 2, C and D). Representative short-axis echocardiography and M-mode images are shown in Figure 2B, and details of echocardiography are shown in Table 2. In addition, AAV2/9-miR-30-Zsgreen treatment reduced the heart weight/tibial length, LV weight/tibial length and LV weight/heart weight ratios in nephrectomized rats (Figure 3, C–E). Correspondingly, cross sections of the left ventricle exhibited thinner LV walls in AAV2/9-miR-30-Zsgreen–injected rats, while WGA staining of the left ventricle demonstrated a decreased cross-sectional area of cardiomyocytes in AAV2/9-miR-30-Zsgreen–treated rats (Figure 2B and Figure 3F).

Then, we performed qPCR to analyze the alterations in the molecular hypertrophic indicators. Two established markers of cardiac hypertrophy, atrial natriuretic peptide (*Anp*) and brain natriuretic peptide (*Bnp*) (38), in the AAV2/9-miR-30-Zsgreen–treated rats were decreased compared with those of AAV2/9-Zsgreen–injected rats (Figure 3, G and H). The expression of adult α–myosin heavy chain (*α-Mhc*) decreased, while the expression of fetal *β-Mhc* increased, which indicates the fetal genes associated with cardiac hypertrophy were reactivated (39). Despite failing to observe the upregulation of *α-Mhc* expression in AAV2/9-miR-30-Zsgreen–treated rats, reactivation of *β-Mhc* was obviously inhibited (Figure 3, I and J). We also tested whether miR-30 can influence cardiomyocyte apoptosis, which has been implicated as a primary mechanism in cardiac remodeling under CKD conditions (40, 41). As expected, AAV2/9-miR-30-Zsgreen treatment significantly attenuated cardiomyocyte apoptosis in SN rats (Supplemental Figure 6, A and B). In brief, these data indicated that miR-30 attenuated the development of cardiac hypertrophy in CKD without influencing CKD progression, and miR-30 should be an indispensable part in CKD-induced LVH.

### Inhibition of endogenous miR-30 in cardiomyocytes causes hypertrophy.

With the aid of miRNA sponge technology (42), we further demonstrated the specific role of miR-30 in cardiomyocytes. We crossed miR-30 sponge transgenic mice that were already produced (37) with cardiomyocyte-specific *Myh6-Cre* transgenic mice to generate double-transgenic (30SP) mice (Supplemental Figure 7A). miR-30 sponge transgenic mice were used as the control group. The 30SP mice lost red fluorescent protein (RFP) expression in cardiomyocytes (Supplemental Figure 7B), and qPCR analysis showed miR-30 sponge expression in the myocardial tissues of 30SP mice (Supplemental Figure 7C). Compared with the control group, 30SP mice displayed normal growth as indicated by the unchanged body weight and tibial length (Supplemental Figure 7, D and E).

**Figure 4:**
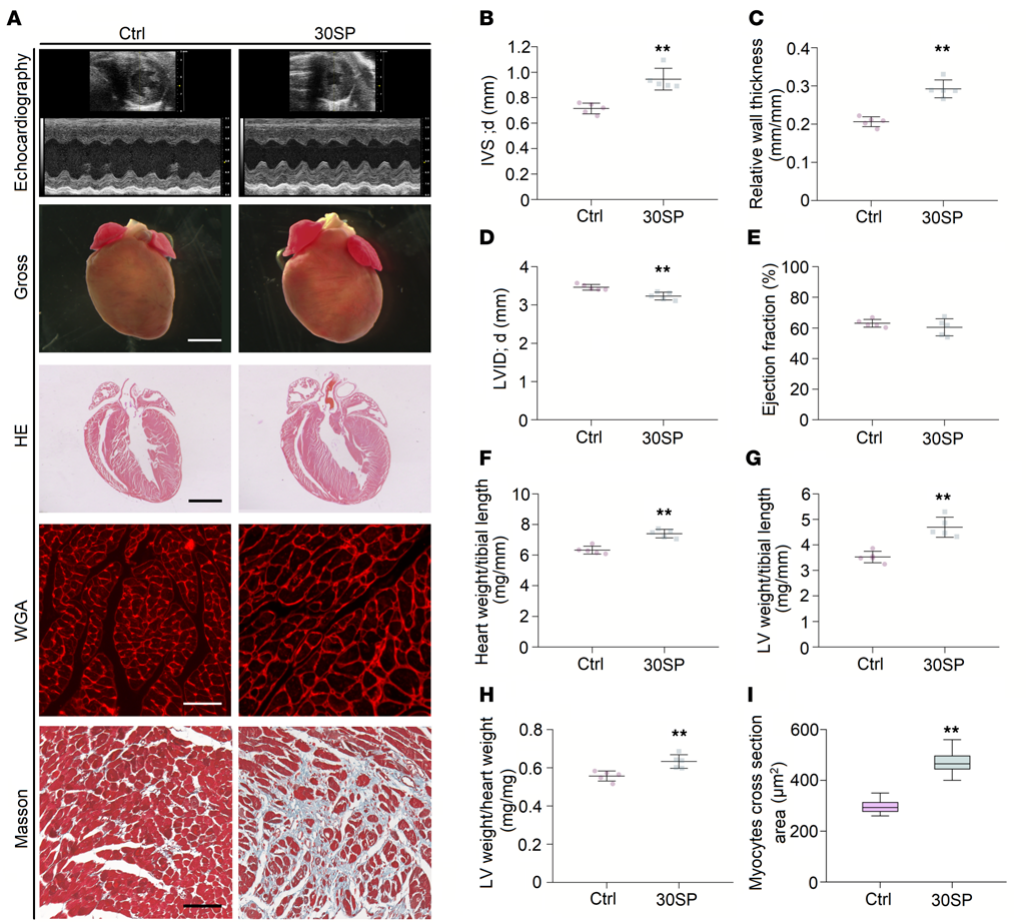
miR-30 knockdown in cardiomyocytes induces LVH. (**A**) Representative echocardiogram, gross (original magnification, ×8; scale bar: 500 μm) and sagittal sections of the heart (hematoxylin and eosin staining; original magnification, ×8; scale bar: 500 μm), and WGA (original magnification, ×400; scale bar: 50 nm) and Masson staining of left ventricle (original magnification, ×400; scale bar: 50 nm). (**B** and **C**) IVS; d and relative wall thickness are significantly increased in 30SP mice. **P < 0.01 compared with values for the control (ctrl), by 2-tailed, unpaired Student’s *t* test. Data are shown as mean ± SD. *n* = 5 mice per group. (**D**) LVID; d is reduced in 30SP mice. **P < 0.01 compared with values for the ctrl, by 2-tailed, unpaired Student’s *t* test. Data are shown as mean ± SD. *n* = 5 mice per group. (**E**) No significant difference in ejection fractions between ctrl and 30SP mice. Compared with values for the ctrl, by 2-tailed, unpaired Student’s *t* test. Data are shown as mean ± SD. *n* = 5 mice per group. (**F**–**H**) 30SP mice manifest obvious increases in the ratios of heart weight to tibial length, LV weight to tibial length, and LV weight to heart weight. **P < 0.01 compared with values for the ctrl, by 2-tailed, unpaired Student’s *t* test. Data are shown as mean ± SD. *n* = 5 mice per group. (**I**) An increased cross-sectional area of cardiomyocytes in 30SP mice. **P < 0.01 compared with values for the ctrl, by 2-tailed, unpaired Student’s *t* test. Data are shown as median and quartiles, as well as the minimum and maximum values of the distribution. *n* = 300 cells per group.

30SP mice developed significant cardiac hypertrophy at 24 weeks after birth, as exhibited by increased LV wall thickness and relative wall thickness (Figure 4, B and C). Moreover, a decreased LV diameter was observed in 30SP mice (Figure 4D). However, LV ejection fraction did not change remarkably (Figure 4E). Figure 4A and Table 3 present representative echocardiography images and detailed echocardiography results, respectively. A representative longitudinal section of the heart from 30SP mice demonstrated LV hypertrophy (Figure 4A). Consistent with the echocardiography data described above, the heart weight/tibial length, LV weight/tibial length, and LV weight/heart weight ratios of 24-week-old 30SP mice were markedly increased, which support LVH in 30SP mice (Figure 4, F–H). Correspondingly, WGA staining showed an increased cross-sectional area in cardiomyocytes from 30SP mice (Figure 4, A and I). All of these observations indicated that inhibition of endogenous miR-30 in cardiomyocytes can directly induce cardiac hypertrophy.

### Inhibition of miR-30 leads to calcineurin activation and pathological hypertrophy.

**Figure 5:**
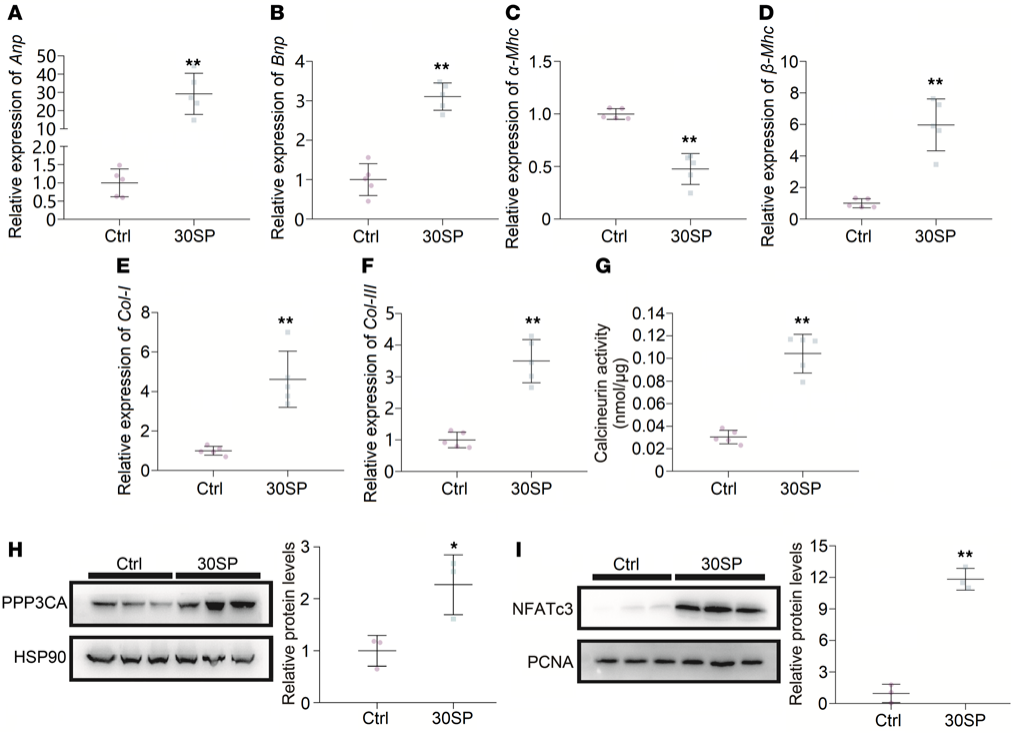
miR-30 knockdown in cardiomyocytes induces pathological hypertrophy and activates the Calcineurin pathway. (**A**–**D**) miR-30 knockdown leads to increased expression of *Anp*, *Bnp*, and *β-Mhc* in the left ventricle, whereas *α-Mhc* is downregulated. Expression levels are normalized by *18S*. **P < 0.01 compared with values for the ctrl, by 2-tailed, unpaired Student’s *t* test. Data are shown as mean ± SD. *n* = 5 mice per group. (**E** and **F**) Upregulated *Col I* and *Col III* mRNA in the left ventricles from 30SP mice. Expression levels are normalized by *18S*. **P < 0.01 compared with values for the ctrl, by 2-tailed, unpaired Student’s *t* test. Data are shown as mean ± SD. *n* = 5 mice per group. (**G**) Cardiac phosphatase activity of Calcineurin strengthened by miR-30 knockdown in 30SP mice. **P < 0.01 compared with values for the ctrl, by 2-tailed, unpaired Student’s *t* test. Data are shown as mean ± SD. *n* = 5 mice per group. (**H** and **I**) Western blotting reveals enhanced PPP3CA expression and nuclear translocation of NFATc3 in 30SP mice. Protein levels are normalized by heat shock protein 90 (HSP90) or proliferation cell nuclear antigen (PCNA). *P < 0.05, **P < 0.01 compared with values for the ctrl, by 2-tailed, unpaired Student’s *t* test. Data are shown as mean ± SD. *n* = 3 independent experiments per group.

We collected LV tissues from 30SP mice to analyze the phenotype of these hypertrophic hearts. Increased expression of hypertrophic genes and interstitial fibrosis are common features of pathological cardiac hypertrophy (7, 43). We performed qPCR to reveal the expression pattern of hypertrophic genes and found that *α-Mhc* expression was decreased, while the expression of *Anp*, *Bnp*, and *β-Mhc* was increased (Figure 5, A–D). Interstitial fibrosis means excessive accumulation of collagen in myocardial interstitium, and Masson staining of myocardial sections of the left ventricle showed obvious cardiac interstitial fibrosis in 30SP mice (Figure 4A). Accordingly, the mRNA levels of type I collagen (*Col I*) and *Col III*, which are both fibrotic markers, markedly increased in 30SP hearts (Figure 5, E and F). Hence, these data demonstrated that suppression of miR-30 in cardiomyocytes can directly result in pathological LVH in vivo.

In our previous study, we found that PPP3CA, PPP3CB, and PPP3R1, all of which are subunits of calcineurin, are direct targets of miR-30 (37). Hence, we speculated that inhibition of miR-30 in cardiomyocytes would result in the activation of the calcineurin pathway, which is considered a critical mediator of cardiac hypertrophy (38). Calcineurin activity assays showed a significantly enhanced phosphatase activity of calcineurin in the myocardium of 30SP mice, which indicated that calcineurin signaling might contribute to this hypertrophic phenotype (Figure 5G). Correspondingly, cardiac *PPP3CA* was upregulated in 30SP mice, and this also confirmed that miR-30 sponge could disinhibit natural targets of miR-30 in cardiomyocytes (Figure 5H). Nuclear factor of activated T cells cytoplasmic 3 (NFATc3) is an indispensable component of Calcineurin-induced cardiac hypertrophy, and it can be dephosphorylated by calcineurin, thus translocating to the nucleus and exerting transcriptional regulatory functions (44). Indeed, Western blotting revealed that the nuclear translocation of NFATc3 was enhanced in 30SP mice (Figure 5I).

**Figure 6:**
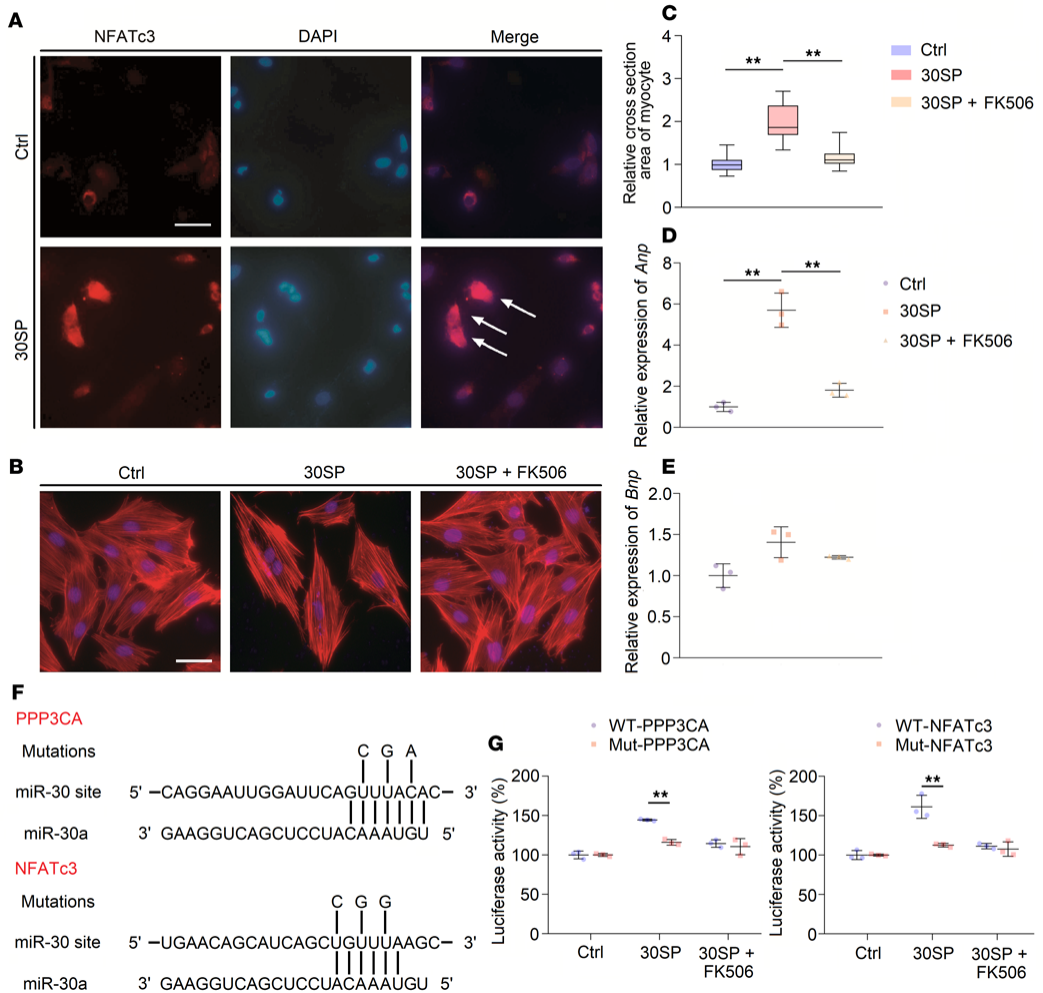
miR-30 knockdown induces cardiomyocyte hypertrophy via Calcineurin signaling. (**A**) miR-30 sponge transfection into H9c2 cells leads to nuclear translocation of NFATc3. White arrows indicate NFATc3 gathers in nuclei (original magnification, ×400; scale bar: 50 nm). Ang II, angiotensin II; IS, indoxyl sulfate. (**B** and **C**) miR-30 sponge transfection results in H9c2 hypertrophy, and this effect is inhibited by FK506 (original magnification, ×400; scale bar: 50 nm). **P < 0.01 compared with values indicated by the dashed line, by 1-way ANOVA test. Tukey’s multiple comparisons test was used for multiple comparison. Data are shown as median and quartiles, as well as the minimum and maximum values of the distribution. *n* = 40 cells per group, in triplicated experiments. (**D**) miR-30 sponge transfection upregulates expression of *Anp*, while this can be suppressed by FK506. Expression levels are normalized by *18S*. **P < 0.01 compared with values indicated by the dashed line, by 1-way ANOVA test. Tukey’s multiple comparisons test was used for multiple comparison. Data are shown as mean ± SD of 3 independent experiments. (**E**) miR-30 sponge transfection has no obvious effect in *Bnp* expression. Expression levels are normalized by *18S*. Compared by 1-way ANOVA test. Tukey’s multiple comparisons test was used for multiple comparison. Data are shown as mean ± SD of 3 independent experiments. (**F**) Predicted miR-30a–targeted sites in the 3′-UTRs of *Ppp3ca* and *NFATc3*; mutations of 3 nucleotides as indicated are made for each mutant reporter construct. (**G**) The effects of miR-30 knockdown using the miR-30 sponge on luciferase reporter expression. **P < 0.01 compared with values indicated by the dashed line, by 2-tailed, unpaired Student’s *t* test. Data are shown as mean ± SD of 3 independent experiments.

Subsequently, we verified abovementioned results in vitro. We transfected a miR-30 sponge plasmid into H9c2 cells and found that NFATc3 was distinctly localized in the nuclei of these miR-30 sponge–transfected cells (Figure 6A). Moreover, we found that the size of the miR-30 sponge–transfected cells was increased, and this effect was inhibited by FK506, which is a specific inhibitor of calcineurin (45) (Figure 6, B and C). In support of this finding, the increased expression levels of *Anp* were inhibited by FK506 (Figure 6D), although there were no significant changes in *Bnp* expression (Figure 6E). Both *Ppp3ca* and *Nfatc3* are predicted to be miR-30 targets (37); therefore, we performed luciferase reporter assays to confirm that *Ppp3ca* mRNA and *Nfatc3* mRNA are direct targets of miR-30 in cardiomyocytes. For each sequence, we produced a construct containing the luciferase coding region, followed by either the wild-type (WT) 3′-untranslated region (UTR) or a mutant 3′-UTR (Figure 6F). When cultured H9c2 cells were cotransfected with the miR-30 sponge plasmid, the WT reporter exhibited higher luciferase activity than the mutant reporter (Figure 6G).

Calcineurin signaling seems to play an important role in CKD-induced LVH (35); hence, we tested whether cardiac miR-30 could suppress the activation of calcineurin pathway in the myocardium of CKD rats. CKD robustly upregulated PPP3CA (Supplemental Figure 8A) and promoted the nuclear translocation of NFATc3 (Supplemental Figure 8B) in cardiac tissues, whereas AAV2/9-miR-30-Zsgreen treatment significantly attenuated this calcineurin signaling activation (Supplemental Figure 8, A and B). Collectively, these results suggested that miR-30 downregulation–induced cardiac hypertrophy is mediated by calcineurin signaling and that miR-30 suppression is closely related to calcineurin activation in CKD-induced LVH.

### CKD-related prohypertrophic factors mediate cardiac miR-30 suppression.

**Figure 7:**
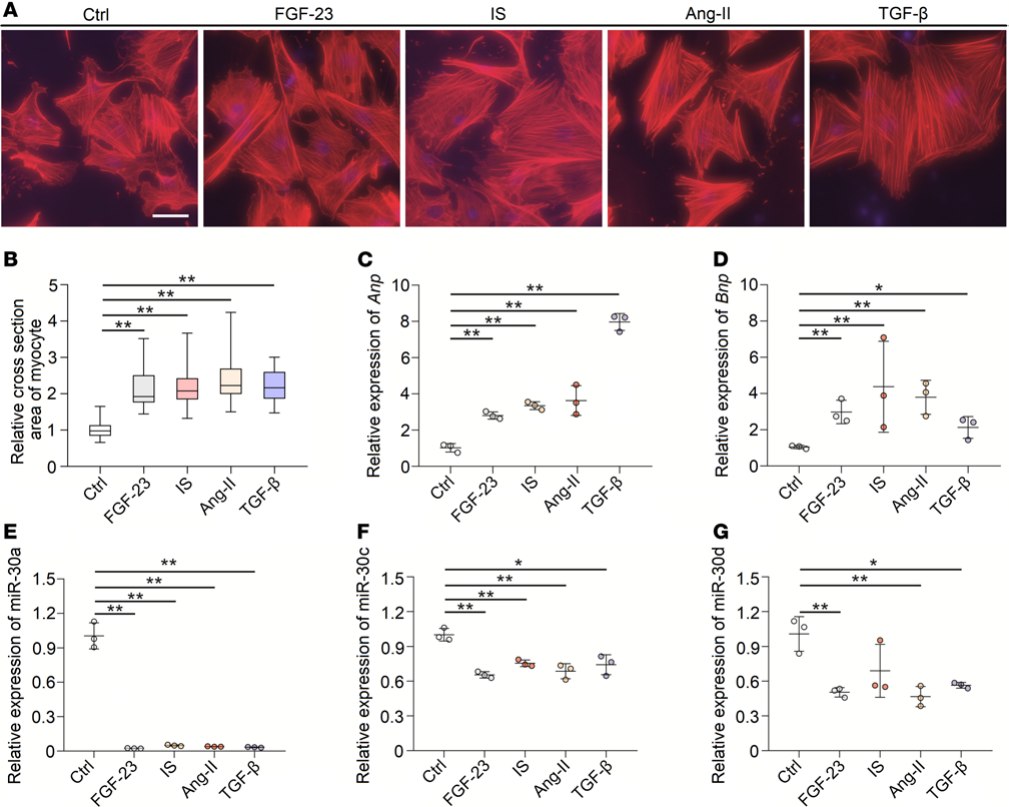
Hypertrophic stimuli suppress miR-30 expression in vitro. (**A**) Representative fluorescein-conjugated phalloidin staining of the NRVMs (original magnification, ×400; scale bar: 50 nm). (**B**) Incubation of FGF-23, IS, Ang II, and TGF-β demonstrates prominent hypertrophy. **P < 0.01 compared with values indicated by the dashed line, by Kruskal-Wallis test. Dunn’s multiple comparisons test was used for multiple comparison. Data are shown as median and quartiles, as well as the minimum and maximum values of the distribution. *n* = 40 cells per group; in triplicated experiments. (**C** and **D**) FGF-23, IS, Ang II, and TGF-β upregulate *Anp* and *Bnp* in NRVMs. Expression levels are normalized by *18S*. *P < 0.05 and **P < 0.01 compared with values indicated by the dashed line, by 1-way ANOVA test. Tukey’s multiple comparisons test was used for multiple comparison. Data are shown as mean ± SD of 3 independent experiments. (**E**–**G**) FGF-23, IS, Ang II, and TGF-β suppress miR-30 expression in NRVMs. Expression levels are normalized by *U6*. *P < 0.05 and **P < 0.01 compared with values indicated by the dashed line, by 1-way ANOVA test. Tukey’s multiple comparisons test was used for multiple comparison. Data are shown as mean ± SD of 3 independent experiments.

LVH can be induced by various detrimental factors in CKD, such as phosphorus metabolism disorder, uremic toxins, RAS activation, and inflammation (10). Thus, we speculated that these factors may suppress miR-30 expression and that miR-30 partially mediates such factor-induced cardiac hypertrophy. We treated neonatal rat ventricular myocytes (NRVMs) with FGF-23 (46), indoxyl sulfate (IS) (47), angiotensin II (Ang II) (48), and TGF-β (49), which are all related to CKD-induced LVH. As expected, FGF-23, IS, Ang II, and TGF-β stimulated cardiomyocyte hypertrophy (Figure 7, A and B) and upregulated *Anp* and *Bnp* in these cells (Figure 7, C and D). Importantly, these prohypertrophic factors also repressed miR-30 expression in NRVMs (Figure 7, E–G).

**Figure 8:**
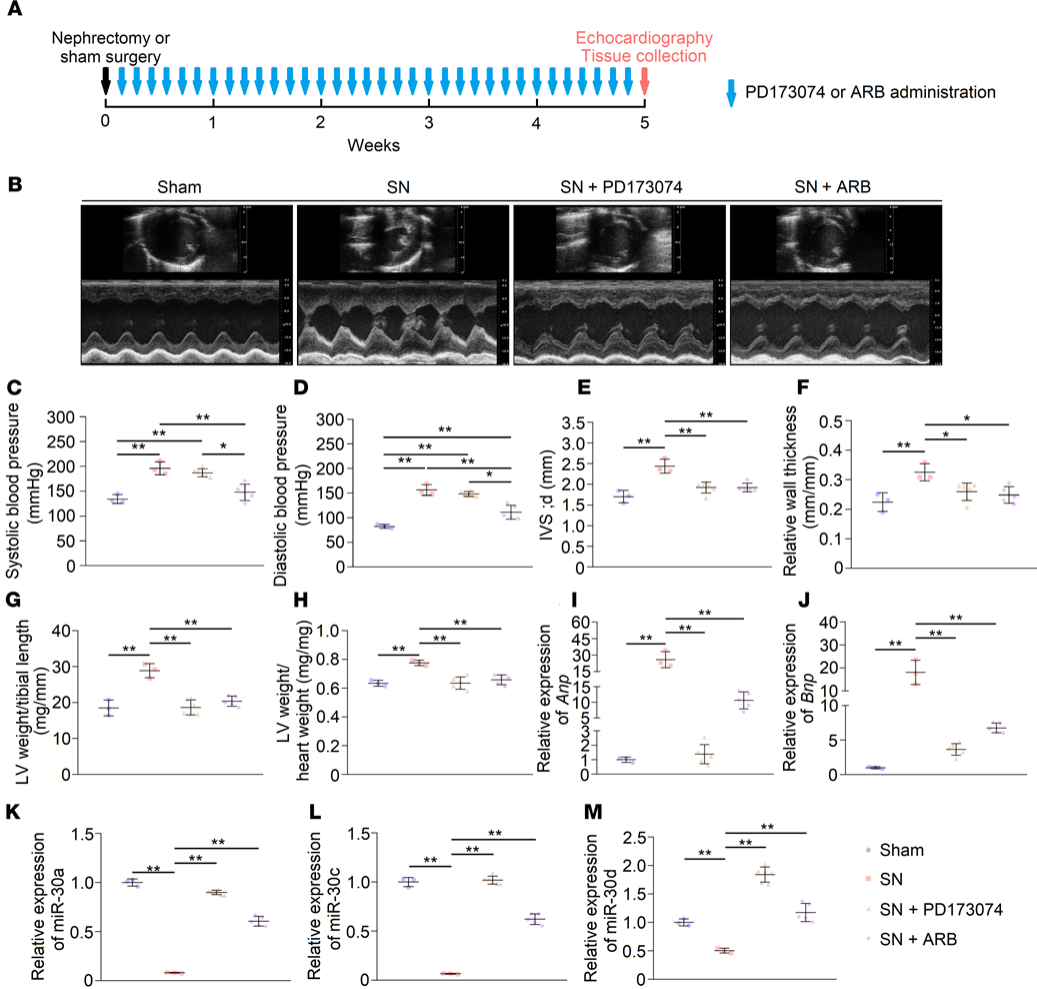
Blocking receptors for FGF-23 and Ang II relieves cardiac miR-30 suppression in CKD. (**A**) Schematic diagram for PD173074 and ARB injection and efficacy observation. (**B**) Representative short-axis echocardiography and M-mode images. (**C** and **D**) PD173074 has no significant effect on blood pressures in CKD rats, whereas ARB lowers blood pressures. *P < 0.05 and **P < 0.01 compared with values indicated by the dashed line, by 1-way ANOVA test. Tukey’s multiple comparisons test was used for multiple comparison. Data are shown as mean ± SD. *n* = 3 to 6 rats per group. (**E**–**J**) PD173074 and ARB both mitigate LVH and suppress upregulation of *Anp* and *Bnp* in CKD rats. Expression levels are normalized by *18S*. *P < 0.05 and **P < 0.01 compared with values indicated by the dashed line, by 1-way ANOVA test. Tukey’s multiple comparisons test was used for multiple comparison. Data are shown as mean ± SD. *n* = 3 to 6 rats per group. (**K**–**M**) PD173074 and ARB inhibit downregulation of cardiac miR-30. Expression levels are normalized by *U6*. **P < 0.01 compared with values indicated by the dashed line, by 1-way ANOVA test. Tukey’s multiple comparisons test was used for multiple comparison. Data are shown as mean ± SD. *n* = 3 to 6 rats per group.

As prohypertrophic factors, FGF-23 and Ang II are 2 major contributors to LVH progression in CKD; therefore, we speculated that these factors also result in cardiac miR-30 suppression under CKD conditions. To test this hypothesis, we treated SN rats with the pan-FGF receptor inhibitor PD173074 or angiotensin receptor blocker (ARB) once daily for 5 weeks (Figure 8A). PD173074 and ARB both had no effects on serum creatinine and urea nitrogen levels in CKD rats (Supplemental Figure 9, A and B), whereas ARB lowered the blood pressures in nephrectomized rats (Figure 8, C and D). Consistent with previous studies (46, 48), both PD173074 and ARB ameliorated LVH in SN rats (Figure 8, E–H). Representative short-axis echocardiography and M-mode images are shown in Figure 8B, and echocardiography measurements are shown in Table 4. In addition, both PD173074 and ARB suppressed the upregulation of *Anp* and *Bnp* in CKD rats (Figure 8, I and J). As expected, PD173074 and ARB restored downregulated cardiac miR-30 in CKD (Figure 8, K–M). However, pressure overload also leads to miR-30 downregulation in myocardium (28); thus we could not rule out that ARB-induced miR-30 rescue is mediated by blood pressures decreasing.

### miR-30 inhibits FGF-23–induced cardiac hypertrophy.

**Figure 9:**
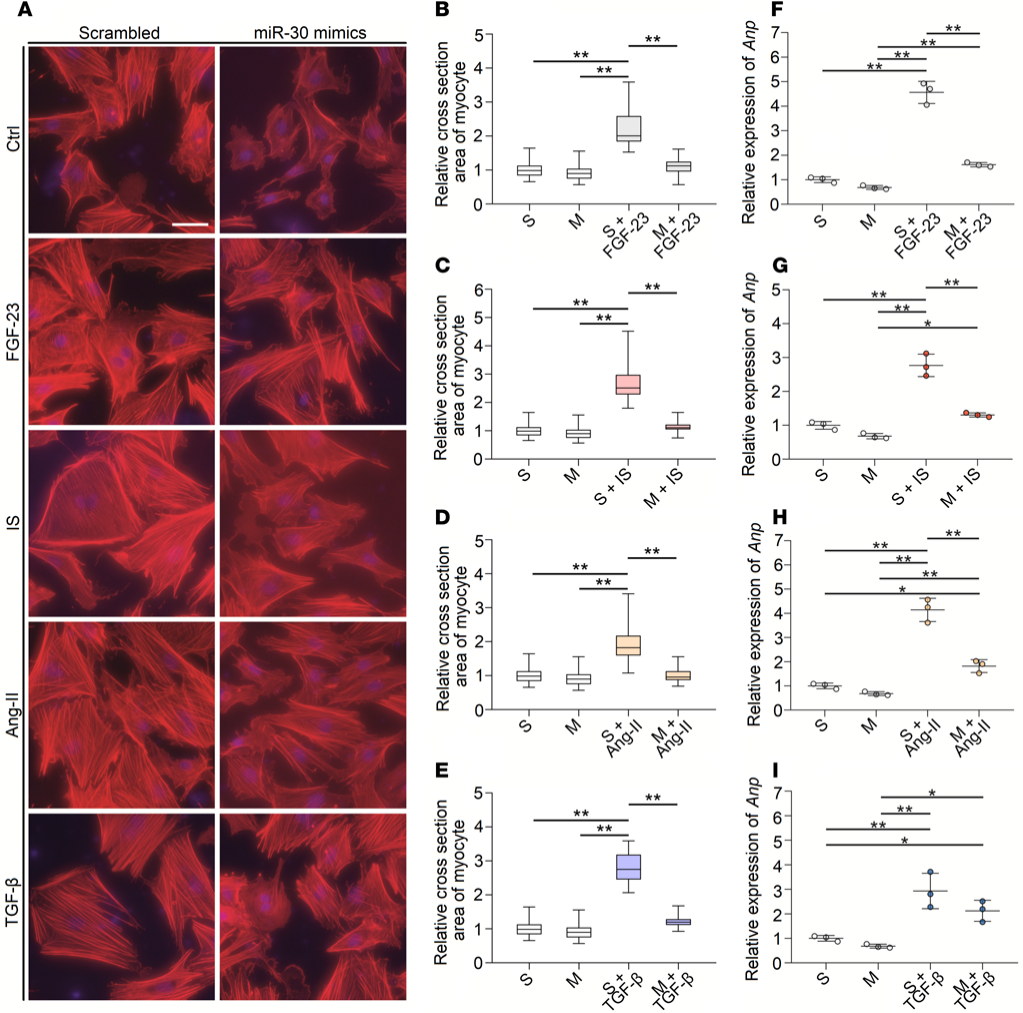
miR-30 inhibits FGF-23–, IS-, Ang II–, and TGF-β–induced cardiomyocyte hypertrophy in vitro. (**A**) Representative fluorescein-conjugated phalloidin staining of the NRVMs (original magnification, ×400; scale bar: 50 nm). (**B**–**E**) miR-30 reduces relative cross-sectional area of prohypertrophic stimuli-treated cardiomyocytes. **P < 0.01 compared with values indicated by the dashed line, by Kruskal-Wallis test. Dunn’s multiple comparisons test was used for multiple comparison. Data are shown as median and quartiles, as well as the minimum and maximum values of the distribution. *n* = 40 cells per group, in triplicated experiments. (**F**–**I**) In addition to TGF-β, miR-30 inhibits the upregulation of *Anp* in prohypertrophic stimuli-treated cardiomyocytes. Expression levels are normalized by *18S*. *P < 0.05 and **P < 0.01 compared with values indicated by the dashed line, by 1-way ANOVA test. Tukey’s multiple comparisons test was used for multiple comparison. Data are shown as mean ± SD of at least 3 independent experiments.

Based on the results above, we speculated that miR-30 can blunt prohypertrophic factor-induced cardiomyocyte hypertrophy, hence mitigating LVH in CKD. This hypothesis was tested in vitro first; we transfected NRVMs with miR-30 mimics and treated these transfected cells with FGF-23, IS, Ang II, and TGF-β. Compared with scrambled miR-transfected cells, miR-30 mimic–transfected cells exhibited significantly less hypertrophy (Figure 9, A–E) and less upregulated *Anp* expression (except TGF-β) (Figure 9, F–I). It is worth mentioning that FGF-23– and Ang II–induced cardiomyocyte hypertrophy are mediated by calcineurin signaling (46, 50), which means that miR-30–suppressed cardiomyocyte hypertrophy induced by FGF-23 and Ang II are mediated by calcineurin pathway inactivation. Western blotting revealed that miR-30 mimics inhibited nuclear translocation of NFATc3 in FGF-23–treated NRVMs, despite the expression of PPP3CA being unaffected (Supplemental Figure 10, A and B).

**Figure 10:**
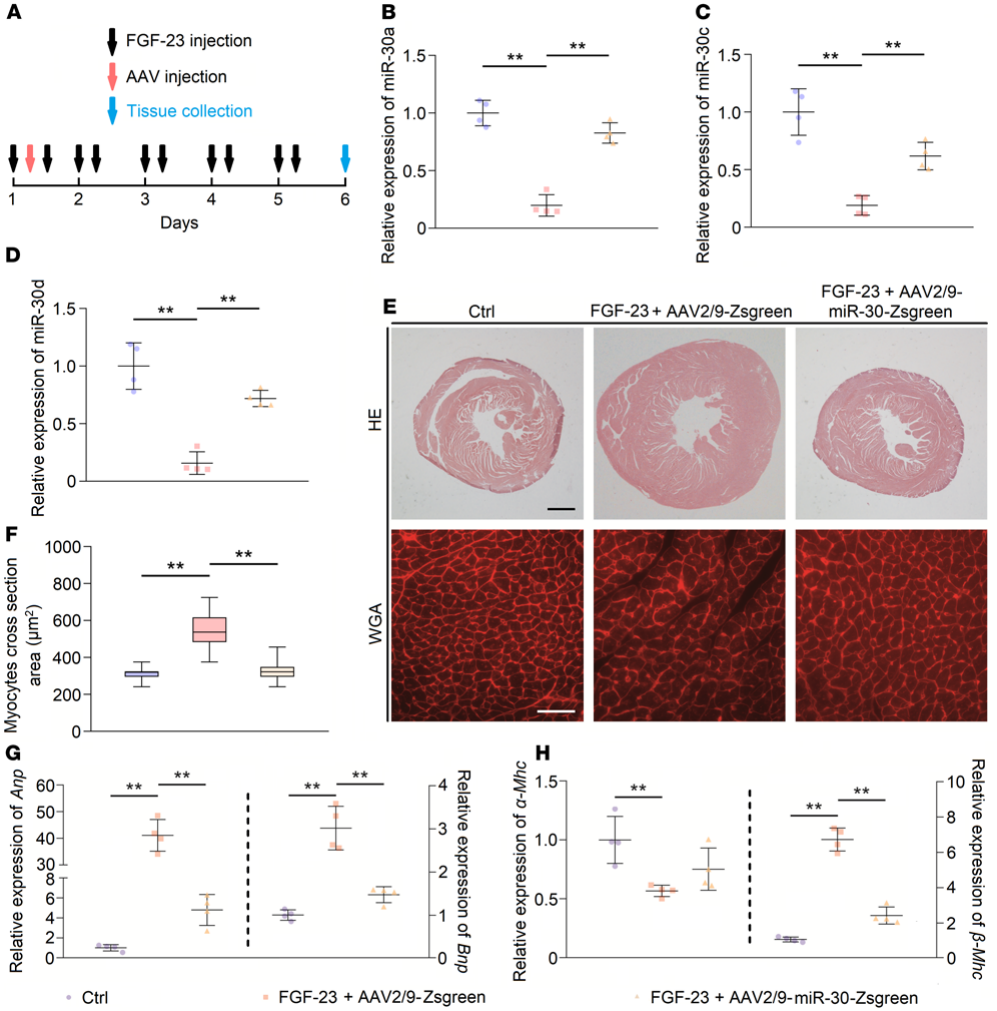
miR-30 inhibits FGF-23–induced cardiac hypertrophy in vivo. (**A**) Schematic diagram for FGF-23 and AAV2/9 injection. (**B**–**D**) AAV2/9-miR-30-Zsgreen treatment rescues the downregulation of cardiac miR-30 in FGF-23–treated mice. Expression levels are normalized by *U6*. **P < 0.01 compared with values indicated by the dashed line, by 1-way ANOVA test. Tukey’s multiple comparisons test was used for multiple comparison. Data are shown as mean ± SD. *n* = 4 mice per group. (**E**) Representative cross sections (hematoxylin and eosin staining; original magnification, ×20; scale bar: 200 μm) and WGA staining (original magnification, ×400; scale bar: 50 nm) of myocardium. (**F**) miR-30 rescue significantly reduces FGF-23–induced cardiomyocyte hypotrophy. **P < 0.01 compared with values indicated by the dashed line, by Kruskal-Wallis test. Dunn’s multiple comparisons test was used for multiple comparison. Data are shown as median and quartiles, as well as the minimum and maximum values of the distribution. *n* = 240 cells per group. (**G** and **H**) miR-30 mitigates upregulated hypertrophic indicators in FGF-23–treated mice, despite having no significant effect on *α-Mhc*. Expression levels are normalized by *18S*. **P < 0.01 compared with values indicated by the dashed line, by 1-way ANOVA test. Tukey’s multiple comparisons test was used for multiple comparison. Data are shown as mean ± SD. *n* = 4 mice per group.

Subsequently, we tested whether FGF-23 could downregulate cardiac miR-30 and whether miR-30 could inhibit FGF-23–induced cardiac hypertrophy in vivo. We treated male C57BL/6 mice with recombinant FGF-23 via tail vein injection, while the dose and frequency were based on those used in a published report by Faul et al. (46). After the first FGF-23 injection, we administrated 10^11^ vg AAV2/9-Zsgreen or 10^11^ vg AAV2/9-miR-30-Zsgreen to these mice by subclavian vein injection (Figure 10A). After 5 days of continuous FGF-23 injection, mice were sacrificed, and heart tissues and blood were collected. Circulating levels of FGF-23 were significantly increased compared with those of saline-injected mice, whereas AAV injections had no influence on circulating FGF-23 (Supplemental Figure 11A). Consistent with the results of Faul et al. (46), FGF-23 injection resulted in significant LVH and cardiomyocyte hypertrophy (Figure 10, E and F), as well as miR-30 downregulation. As expected, AAV2/9-miR-30-Zsgreen administration rescued miR-30 repression (Figure 10, B–D) and attenuated LVH and cardiomyocyte hypertrophy in FGF-23–injected mice (Figure 10, E and F). Consistent with these results, miR-30 rescue suppressed the expression pattern of hypertrophic genes (except *α-Mhc*) (Figure 10, G and H).

In a previous report, FGF-23–induced cardiac hypertrophy was mediated by the calcineurin pathway (46). Thus, we speculated that miR-30 could suppress FGF-23–induced hypertrophy by inactivating calcineurin signaling. Indeed, miR-30 blunted the nuclear translocation of NFATc3 (Supplemental Figure 11C) in FGF-23–treated mice, although it did not affect the expression of PPP3CA (Supplemental Figure 11B).

## Discussion

Predialysis or dialysis CKD patients regularly present with marked LVH (51–53), which is a pathological basis of CKD-related cardiovascular complications. However, much is still unknown about the pathophysiological mechanism of CKD-induced LVH. Importantly, growing evidence has demonstrated that miRNAs are broadly involved in cardiac hypertrophy and may act as mediators of hypertrophy progression (12). miR-30 is highly expressed in cardiac tissues and is suppressed under hypertrophic conditions, indicating its vital function in the heart (21–23). Thus, we were prompted to explore the potential role of cardiac-specific miR-30 in the occurrence and development of CKD-induced LVH.

miR-30 is abundantly expressed in cardiac tissues under physiological conditions (21–23), and its expression level suggests a vital role of this miRNA family. After nephrectomy, cardiac hypertrophy and myocardial miR-30 suppression were observed (Figure 1J), primarily indicating the relationship between miR-30 inhibition and cardiac hypertrophy progression in CKD. Based on these results, we speculated that miR-30 could act as an antihypertrophic factor. Indeed, cardiomyocyte-specific miR-30 rescued mitigated hypertrophic conditions in CKD rats (Figure 2 and Figure 3).

Several pathways are considered to be involved in CKD-induced LVH. For instance, mitogen-activated protein kinase (MAPK) signaling can be induced by hemodynamic stress or Ang II stimulation (54, 55). Di Marco et al. (35) identified that the calcineurin-dependent pathway serves as an important mediator of cardiac hypertrophy in CKD conditions. Unlike other prohypertrophic signaling pathways, the calcineurin/NFATc3 pathway participates in pathological, but not physiological, cardiac hypertrophy (38, 44, 56); thus, it may manifest more important significance in CKD-induced LVH. Coincidently, our previous study showed that miR-30 regulates calcineurin/NFATc3 pathway in podocytes (37). According to these results, we hypothesized that miR-30 regulates cardiac hypertrophy in CKD via the calcineurin/NFATc3 pathway. Through miRNA sponge technology, we demonstrated that inhibition of endogenous miR-30 in cardiomyocytes not only directly leads to pathological cardiac hypertrophy but also enhances the phosphatase activity of calcineurin and promotes nuclear translocation of NFATc3 (Figure 4 and Figure 5). In addition, miR-30 sponge plasmids that were transfected into cardiomyocytes also showed enlarged myocytes (Figure 6B) and increased concentrations of NFATc3 in nuclei (Figure 6A), and cardiomyocyte hypertrophy was attenuated by a specific calcineurin inhibitor, FK506 (Figure 6, B–E). Importantly, *Ppp3ca* mRNA and *Nfatc3* mRNA are both direct targets of miR-30 (Figure 6, F and G). Furthermore, miR-30 blunted calcineurin signaling in myocardium from CKD rats and FGF-23–treated mice (Supplemental Figure 8 and Supplemental Figure 11). Thus, we concluded that calcineurin/NFATc3 connects miR-30 with cardiac hypertrophy, and miR-30 downregulation is essential for CKD-induced LVH.

Although studies have revealed that miR-30 is expressed in cardiac fibroblasts and is involved in cardiac remodeling (25, 57), our study, and others, demonstrated that miR-30 can act in cardiomyocytes to regulate cardiac remodeling. We provided several points to support this. First, hypertrophic stimuli suppressed miR-30 expression in cardiomyocytes in vitro (Figure 7), and this effect was attenuated by miR-30 mimic transfection (Figure 9). Second, we chose a cardiomyocyte-specific cTnT promoter to control the expression of miR-30 in AAV2/9-miR-30-Zsgreen (Supplemental Figure 3); therefore, AAV2/9-miR-30-Zsgreen-injected CKD rats showed reduced cardiac hypertrophy, suggesting that cardiomyocyte-expressed miR-30 is directly responsible for this effect. Third, specifically inhibited endogenous miR-30 in cardiomyocytes could directly induce cardiac hypertrophy (Supplemental Figure 7A). On the other hand, it will be interesting to observe the potential role of cardiac fibroblast-specific miR-30 in CKD-induced LVH. Together, current investigations emphasize that miR-30 in cardiomyocytes is highly involved in cardiac hypertrophy under CKD conditions.

Notably, miR-30 knockdown was only induced in cardiomyocytes, whereas these mice demonstrated not only LVH but also significant cardiac fibrosis (Figure 4A). Activated NFAT regulates the fibrosis-related gene expression in smooth muscle cells and fibroblasts, including *Acta2*, *Col III*, and *Mrtfa* (58, 59), whereas cardiomyocyte miR-30 knockdown should not directly result in calcineurin/NFAT activation in cardiac fibroblasts. Based on our results, the 30SP model can be considered a model of “cardiomyocyte-specific overexpression of calcineurin,” while previous studies have revealed that cardiomyocyte-specific overexpression of calcineurin promotes cardiac fibrosis (38, 60, 61). We believe that cardiomyocyte-fibroblast communication is the key to elucidating the fibrotic phenotype in these mice. Fontes et al. (61) found that calcineurin overexpression in cardiomyocytes reduces the expression of connexin 43, which may interrupt cardiomyocyte-fibroblast communication and trigger enhanced fibroblast activities and fibrosis in the myocardium (62). Correspondingly, cardiomyocyte-specific overexpression of calcineurin leads to an increase in collagen gene expression in the myocardium (62). Most recently, Li et al. (63) demonstrated that miR-30d in cardiomyocytes may regulate fibroblasts’ activation via targeting *integrin* *α5*, and cardiomyocytes can release miR-30d–containing extracellular vesicles to inhibit fibroblast proliferation and activation, thus suppressing myocardium fibrosis in ischemic heart failure. Hence, miR-30 downregulation in cardiomyocytes may indirectly and directly activate fibroblasts and promote cardiac fibrosis. Moreover, it would be interesting to investigate whether miR-30 suppression in cardiomyocytes can release profibrotic factors, such as TGF-β, Ang II, and FGF-23, to activate fibroblasts and promote fibrosis.

Several pathogenic stimuli may inhibit the expression of myocardial miR-30 in CKD. Existing studies have revealed that some prohypertrophic factors, such as oxidative stress (31) or hemodynamic disorders (28), induce a decrease in myocardial miR-30. Thus, we surmised that prohypertrophic stimuli should be possible factors to repress the expression of myocardial miR-30. Wang et al. (10) concluded that some other detrimental factors, such as FGF-23, uremic toxin, Ang II and TGF-β, are involved in CKD-induced cardiac hypertrophy, and our results showed that treating cardiomyocytes with such stimuli can reduce miR-30 expression (Figure 7, E–G). Furthermore, FGF-23–treated mice exhibited cardiac miR-30 suppression as well (Figure 10, B–D). Conversely, miR-30 mimic transfection attenuated cardiomyocyte hypertrophy that was induced by FGF-23, uremic toxin, Ang II, and TGF-β (Figure 9). Notably, FGF-23 receptor and Ang II receptor blockade rescued cardiac miR-30 expression in CKD rats (Figure 8, K–M), despite Ang II receptor blockade also reducing blood pressures (Figure 8, C and D). These results suggested that miR-30 downregulation may function as a common mediator of cardiac hypertrophy in CKD.

Our findings suggest that miR-30 exerts an antihypertrophic effect in cardiomyocytes via the calcineurin/NFATc3 cascade. However, not all stimuli induce hypertrophy directly through calcineurin-related signaling, such as IS, tumor necrosis factor–α (TNF-α), and TGF-β. Previous studies have shown that IS induces cardiac hypertrophy by enhancing the generation of ROS and then regulates several hypertrophy-related signaling pathways, including the MAPK (47), AMPK (64), and NF-κB pathways (65). It has been shown that miR-30a-5p ameliorates oxidative stress by suppressing MAPK signaling in microglial cells; thus, miR-30 may inhibit IS-induced hypertrophy via the same mechanism (67). In addition, miR-30d prevents TNF-α–induced cardiomyocyte apoptosis by targeting MAPK kinase kinase kinase 4 (33). Xu et al. (67) reported that miR-30c suppresses the TGF-β cascade by targeting TGF-β receptor II, which provides a possible explanation for miR-30 suppressing TGF-β–induced hypertrophy. Collectively, miR-30 exerts its function by simultaneously regulating distinctive pathways, which means it is suited to protect cardiomyocytes from concurrent multiple detrimental factors.

Of note, miRNAs can not only bind to the 3′-UTR of mRNA to inhibit mRNA translation or even cut mRNA but also bind to the 5′-UTR of mRNA to activate translation (68). We further speculated that miR-30 may be able to inhibit the signaling that interferes with normal cardiomyocyte functions and activates the cascades that maintain normal cardiomyocyte functions. Downregulation of miR-30 may disturb cardiomyocyte homeostasis, while apoptosis and hypertrophy are reflections of this disturbance. This hypothesis suggests that the function of miR-30 in cardiomyocytes needs further investigation, which is important for providing a solid foundation for its clinical application.

**Figure 11:**
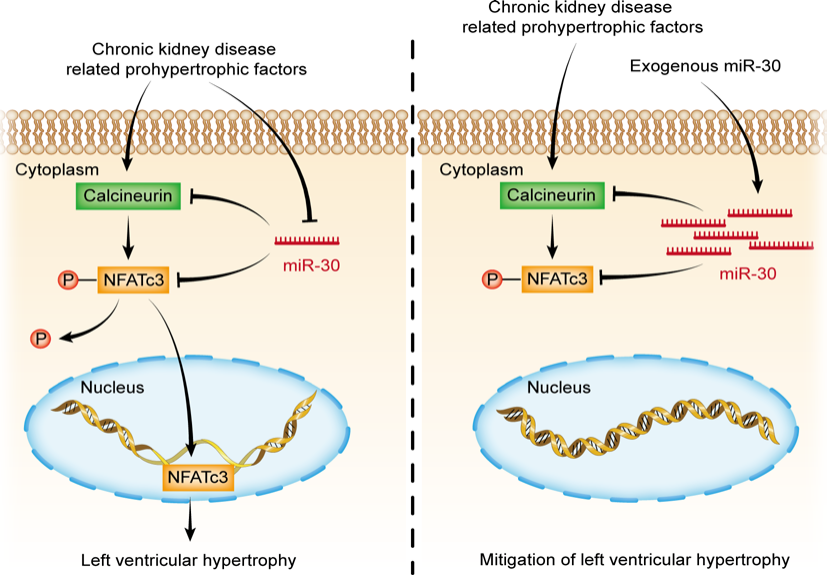
miR-30 rescue mitigates CKD-induced LVH via inactivating Calcineurin signaling.

In summary, we extended our comprehension of myocardial miR-30 in CKD-induced cardiac hypertrophy. We showed myocardial miR-30 suppression in CKD rats, while cardiac-specific miR-30 transfection attenuated the cardiac hypertrophy induced by SN. Importantly, genetic knockdown of miR-30 in cardiomyocytes directly led to cardiac hypertrophy. We further confirmed that downregulation of miR-30 facilitates the activation of the calcineurin/NFATc3 signaling. Above all, miR-30 suppression may result in cardiac hypertrophy via calcineurin/NFATc3 activation in LVH (Figure 11). We believe these data not only uncover a novel mechanism of CKD-induced LVH but also hint at a potential therapeutic target for CKD patients with cardiac hypertrophy.

## Methods

### Animals.

CKD model was induced in 200 g male Sprague-Dawley rats (Sino-British SIPPR/BK Lab Animal Ltd.) via SN method as previously described (46). Rats that were used to observe myocardial miR-30 alterations under CKD conditions were randomized into 6 groups, with 6 animals per group: 3 groups under sham nephrectomy and 3 groups under SN. Rats that were used to observe the protection of miR-30 were randomized into 3 groups, with 6 animals per group; the 10^11^ vg AAV2/9-miR-30-Zsgreen or 10^11^ vg AAV2/9-Zsgreen were dissolved in sterile saline and injected in the jugular veins of nephrectomized rats 3 days after surgery. Rats that were used to observe the inhibition of FGF-23 and Ang II were randomized into 4 groups, with 3–6 animals per group: sham nephrectomy or SN plus 5-week daily intraperitoneal injections of vehicle (PBS) or PD173074 (MilliporeSigma) at 1 mg/kg/d and a gavage of Ang II receptor blocker valsartan (Novartis) at 10 mg/kg/d once daily for 5 weeks.

Conditional miR-30 sponge transgenic mice on the C57BL/6 background were generated by the Model Animal Research Center at Nanjing University. A specific description of miR-30 sponge mice is presented in our previous study (37). Briefly, a miR-30 sponge sequence contained 11 miR-30 cognate sites and was synthesized and inserted downstream of the enhanced green fluorescent protein (eGFP) coding region of the vector pCAG-loxP-RFP-STOP-loxP-eGFP (Model Animal Research Center of Nanjing University). Conditional miR-30 sponge transgenic mice were crossed with *Myh6-Cre* mice (37, 69) (generated by Model Animal Research Center of Nanjing University) to excise the STOP element and result in cardiomyocyte-specific miR-30 sponge expression.

For the FGF-23 treatment, 12-week-old male C57BL/6 mice (Sino-British SIPPR/BK Lab Animal Ltd.) were randomly divided into 3 groups, with 4 animals per group. FGF-23 (Research & Diagnostics Systems, Bio-Techne) and pan-FGF receptor blocker PD173074 (MilliporeSigma) were dissolved in PBS; the mice were administered FGF-23 (40 μg/kg) or PBS via tail vein injection twice daily with 8 hours between injections for 5 consecutive days and an intraperitoneal injection of PD173074 (1 mg/kg) once daily for the same duration. The 10^11^ vg AAV2/9-miR-30-Zsgreen or 10^11^ vg AAV2/9-Zsgreen were dissolved in sterile saline and injected in the jugular veins of C57/B6 mice after the first FGF-23 injection. On the morning of the sixth day, 16 hours after the final tail vein injections, the animals were sacrificed, and the blood and heart tissues were collected.

### Isolation and culture of NRVMs.

We performed the isolation according to Faul et al. (46) and made some modifications. Briefly, the apex of hearts from 1- to 3-day-old Sprague-Dawley rats were harvested and minced in HBSS, and tissues were digested with 0.06% trypsin overnight. On the second day, tissues were further digested with collagenase (MilliporeSigma). After standing still, the supernatant was drawn through the cell screen and filtered into a 50 mL centrifuge tube. We transferred the cell suspension to a 15 mL centrifuge tube and centrifuged (800–1000*g*, 5 minutes, room temperature) to terminate the digestion. After centrifugation, the supernatant was discarded and resuspended with α-MEM medium (Wisent) containing 20% fetal bovine serum (Gibco, Thermo Fisher Scientific) and 1% penicillin/streptomycin. The cardiomyocytes were transfected with 50 nmol/L miR-30 mimics or 50 nmol/L scrambled miR via FuGENE HD Transfection Reagent (Promega), and 4 hours later, these transfected cells were treated with 25 ng/mL FGF-23 (Research & Diagnostics Systems, Bio-Techne), 10^–6^ mol/L Ang II (MilliporeSigma), 100 μmol/L IS (MilliporeSigma), and 10 ng/mL TGF-β (Research & Diagnostics Systems, Bio-Techne), both for 24 hours under an atmosphere of 5% (v/v) CO_2_ in air at 37°C.

### Culture and transfection of H9c2 cells.

The rat embryonic heart–derived H9c2 cells were obtained from American Tissue Culture Collection (ATCC). The H9c2 cells were cultured in DMEM (Gibco, Thermo Fisher Scientific) supplemented with 10% fetal bovine serum (Gibco, Thermo Fisher Scientific) and grown under an atmosphere of 5% (v/v) CO_2_ in air at 37°C. The transient transfection was accomplished by FuGENE HD Transfection Reagent (Promega), and 4 hours after transfection, H9c2 cells were treated with 1 μmol/L FK506 (Fujisawa Pharmaceutical Co., Ltd., now Astellas Pharma Inc.) for 24 hours.

### Preparation of miR-30 sponge.

miR-30 sponge, which contains 5 repeats of the cognate sequence of each miR-30 member, was synthesized and subcloned into pUC plasmid. The detailed sequence of miR-30 sponge was described in our previous report (70).

### miR-30–expressing AAV2/9 preparation and jugular vein injection.

AAV2/9-miR-30-Zsgreen and AAV2/9-Zsgreen were generated by HAN BIO. Briefly, FGF-23–treated mice and SN rats were placed in the supine position and were anesthetized with isoflurane. Then, an incision was made in the subcutaneous tissue of the neck and the subclavian vein was located. Subsequently, we punctured the jugular vein via an insulin syringe and slowly injected 10^11^ vg AAV2/9-miR-30-Zsgreen or 10^11^ vg AAV2/9-Zsgreen. After injection, the wounds were closed with sutures.

### Blood pressure.

The blood pressure of each rat was noninvasively determined by a computerized tail-cuff system, BP 2000 Blood Pressure Analysis System (Visitech Systems).

### Echocardiography.

High-resolution echocardiography (30 MHz) was performed on 24-week-old 30SP mice to assess wall and chamber dimensions, LV weight, and LV systolic function using Vevo 2100 Linear Array Imaging (FUJIFILM VisualSonics). Relative wall thicknesses were calculated as the ratio of the IVS; d to LVID; d. M-mode and 2D recordings were obtained under isoflurane anesthesia delivered through a nose mask. To evaluate dynamic changes in the hearts of the rats, echocardiography was performed 1, 3, and 5 weeks after surgery. The AAV-injected rats were observed until 5 weeks after surgery. The PD173074- and ARB-injected rats were observed after 5 weeks of continuous injection.

### Urine collection and serology.

After echocardiography, the rats were placed into a DXL-D rat metabolism cage (Beijing Jiayuan Industrial Technology Co., Ltd.) to collect 24-hour urine samples. Blood was collected from rats at the time of sacrifice via cardiac puncture and centrifuged at 4500*g* and 4°C for 10 minutes. Sera were collected, stored at –80°C, and subsequently analyzed in batches to determine the urea nitrogen and creatinine concentrations. Urine creatinine, serum creatinine, and serum urea nitrogen were measured by automatic biochemistry analyzer (Beckman Coulter). The creatinine clearance rate was calculated by following formula: creatinine clearance rate = (urine creatine × 24-hour urine volume)/(serum creatine × body weight × 0.01).

Blood derived from FGF-23–treated mice was also centrifuged at 4500*g* and 4°C for 10 minutes and was used for circulating FGF-23 assays (KAINOS Laboratories).

### Morphology analysis of mouse and rat tissues.

After urine collection, animals were sacrificed. The kidneys and hearts were isolated and prepared for molecular and histological analyses. Kidneys from rats and hearts from mice were stained with Masson’s dye to observe the fibrosis conditions. Long-axis sections and short-axis sections of hematoxylin and eosin–stained mouse hearts demonstrate the hypertrophic conditions.

### Immunofluorescence and cell surface area analysis.

After treatment, cultured NRVMs and H9c2 cells were fixed in 4% paraformaldehyde for 15 minutes and permeabilized with 0.1% Triton X-100 in PBS, followed by blocking with 5% goat serum in PBS for 1 hour at room temperature; then, cells were incubated with rhodamine-phalloidin (Thermo Fisher Scientific). The mouse monoclonal antibody against NFATc3 (Santa Cruz Biotechnology; catalog sc-8405) was used at 1:1000. Cy3-conjugated goat anti-mouse (Beyotime Biotechnology; catalog A0521) was used as a secondary antibody at 1:500. To visualize nuclei, fixed cells were incubated with DAPI (400 ng/mL in PBS) for 10 minutes. To visualize cellular borders of heart tissues from rats and mice, we fixed tissue with sodium citrate for 20 minutes, followed by blocking with QuickBlock Blocking Buffer for Immunol Staining (Beyotime Biotechnology), then incubated with WGA conjugated to Alexa Fluor 555 (Thermo Fisher Scientific; catalog W32464) at 50 μg/mL. Immunofluorescence images were taken by a DM5000B microscope (Leica). The myocyte cross-sectional area was measured by ImageJ software (NIH, http://rsbweb.nih.gov/ij/). To confirm that AAV2/9 successfully infected rat cardiomyocytes, the paraffin-embedded sections were incubated with primary antibodies against GFP (Abcam; catalog ab6556) and cTnT (Abcam; catalog ab8295). Then, the sections were incubated with an Alexa Fluor 488–conjugated anti-rabbit secondary antibody (Invitrogen, Thermo Fisher Scientific; catalog R37118) or an Alexa Fluor 594–conjugated anti-mouse secondary antibody (Invitrogen, Thermo Fisher Scientific; catalog A21203), and the sections were mounted using Fluoromount (Abcam). The slides were examined using a Zeiss LSM710 confocal microscope or a Leica microscope (DM5000B).

### RNA extraction and quantification.

Cultured H9c2 cells and cardiac tissues of mice and rats were subjected to small RNA or total RNA extraction via mirVana miRNA Isolation Kit (Thermo Fisher Scientific). The mRNA samples’ quantification by qPCR used the kit from TaKaRa Bio. A TaqMan MicroRNA Assay Kit (Thermo Fisher Scientific) was used to quantify miRNA. Relative expression values of mRNA and miRNA were evaluated with the 2^–ΔΔCt^ method using *18S* and *U6* for normalization, respectively.

### Determination of the copy numbers of miR-30 sponge in cardiac tissues.

To determine the copy numbers of miR-30 sponge in cardiomyocytes, we performed absolute quantification via qPCR. The miR-30 sponge plasmid was serially diluted at concentrations of 10^3^, 10^4^, 10^5^, 10^6^, 10^7^, and 10^8^ copies/μL and subjected to qPCR analysis. The standard curve was generated by plotting the Ct values against the concentrations of the samples (copy numbers/μL) and was used to calculate the sponge concentrations in the samples tested.

### Primers.

See Supplemental Table 1 for primer sequences.

### Calcineurin phosphatase activity assays.

The Calcineurin activity of d cardiac tissues of mice was analyzed with a Calcineurin Cellular Activity Assay Kit (Merck-Calbiochem) following the manufacturer’s instructions. Briefly, tissue samples in lysis buffer containing protein inhibitors were spun at 100,000*g* in a centrifuge at 4°C for 45 minutes to extract the soluble proteins. The free phosphate in the extracts was removed by passing the samples through freshly prepared columns containing desalting resin. To measure the Calcineurin phosphatase activity, the concentrations of the protein samples were determined, and equal protein amounts were incubated with the substrate for 30 minutes at 30°C. Liberated phosphate was measured calorimetrically at 620 nm. The Calcineurin activity was calculated by using the standard curve prepared simultaneously in the assays.

### Protein extraction and Western blot analysis.

Nuclear and cytoplasmic proteins of cardiac tissues were extracted through MINUTE Cytoplasmic & Nuclear Extraction Kit (Invent Biotechnologies) following the manufacturer’s instructions. Antibodies against PPP3CA (Proteintech; catalog 13422-1-AP; 1:2000), NFATc3 (Santa Cruz Biotechnology; catalog sc-8405; 1:2000), HSP90 (EnoGene Biotech Co., Ltd.; catalog E1A0013B; 1:500), and PCNA (Proteintech; catalog 10205-2-AP; 1:1000) were used as primary antibodies, and a horseradish peroxidase–conjugated goat anti-rabbit or anti-mouse antibody (TransGen Biotech Co., LTD.; catalog HS101-01 or HS201-01; 1:10,000) was used as a secondary antibody.

### Luciferase reporter assays.

The 3′-UTRs of *Ppp3ca* and *Nfatc3* mRNAs were obtained via PCR using rat genomic DNA as the template. These 3′-UTRs were inserted downstream of the pGL3 promoter (Promega). Site-directed mutagenesis was conducted to generate mutations in the region corresponding to the miR-30 “seed.” The resulting constructs and Firefly-luciferase were transfected into H9c2 by using FuGENE HD Transfection Reagent (Promega). Twenty-four hours later, cell lysates were prepared and subjected to luciferase assays using a Dual-Luciferase Reporter Assay System (Promega). Renilla-luciferase activity was normalized to the corresponding Firefly-luciferase activity.

### TUNEL.

Cardiomyocyte apoptosis was detected by TUNEL assays using the In Situ Cell Death Detection Kit, POD (Roche). Briefly, the LV sections were incubated with proteinase K after deparaffinization. After washing with PBS, the tissues were incubated with TUNEL solution in a moist and dark environment. Then, the tissues were incubated with converter-POD solution in a humidified environment. After washing, TUNEL-positive cells were stained with diaminobenzidine tetrahydrochloride as the chromogen, and the slides were counterstained with Mayer’s hematoxylin (Ourchem, Sinopharm Chemical Reagent Co., Ltd.).

### Statistics.

Data were presented as mean ± SD or median with interquartile range. Normal distribution of data was analyzed by Shapiro-Wilk test. Differences between 2 groups were analyzed using 2-tailed Student’s *t* test or Mann-Whitney *U* test. One-way ANOVA test or Kruskal-Wallis test was used for comparisons between multiple groups, followed by Tukey’s multiple comparisons test or Dunn’s multiple comparisons test for multiple comparison. Statistical analysis was completed by GraphPad Prism 8 software (GraphPad Software, Inc.). *P* < 0.05 and *P* < 0.01 were considered statistically significant and very significant, respectively.

### Study approval.

The Institutional Animal Care and Use Committee at Jinling Hospital approved the use of animals in this study.

## Author contributions

CZ designed the study and reviewed and edited the manuscript. JB and YL performed experiments and wrote the manuscript. QS and WD assisted in animal experiments. RT and XX assisted in in vitro experiments. MZ and LZ offered technical support for histological staining. QZ, HL, and GZ assisted in molecular assays in serum. ZY offered support for cardiac experiments. SS and ZL provided advice on the experimental design and writing of the manuscript.

## Supplementary Material

Supplemental data

## Figures and Tables

**Table 1:**
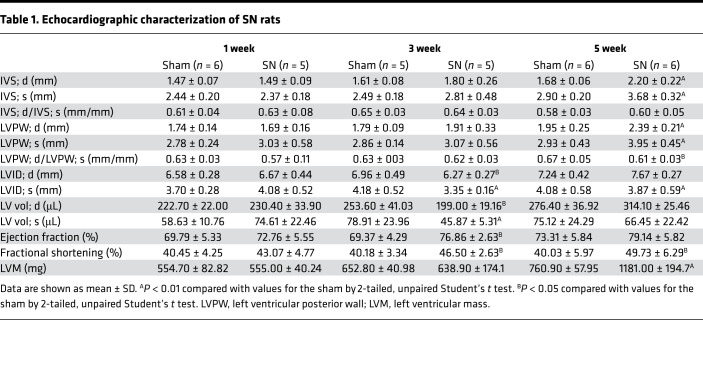
Echocardiographic characterization of SN rats

**Table 2:**
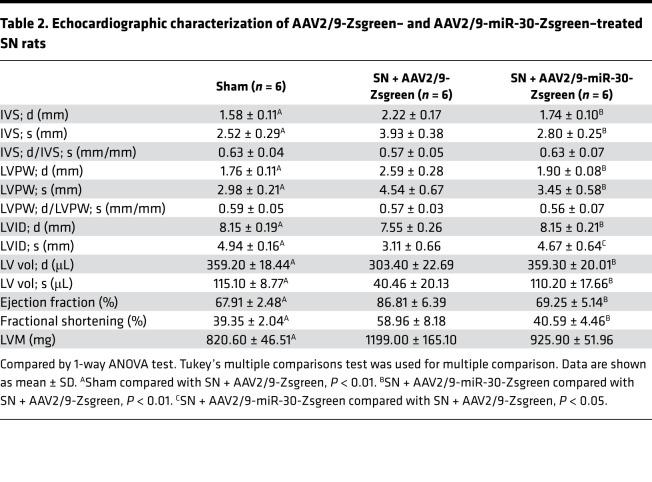
Echocardiographic characterization of AAV2/9-Zsgreen– and AAV2/9-miR-30-Zsgreen–treated SN rats

**Table 3:**
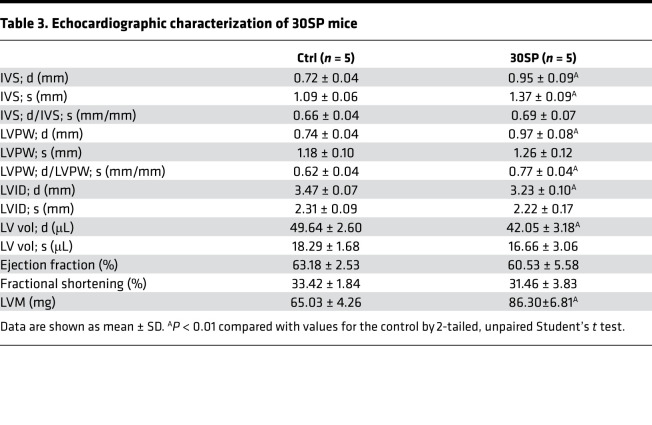
Echocardiographic characterization of 30SP mice

**Table 4:**
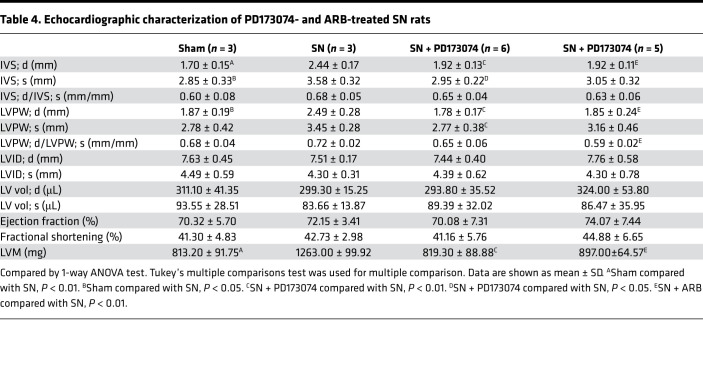
Echocardiographic characterization of PD173074- and ARB-treated SN rats

## Version 1 04/13/2021

In-Press Preview

## Version 2 05/24/2021

Electronic publication
