# Supplementary material for: MicroRNA-30 regulates left ventricular hypertrophy in chronic kidney disease
Source: JCI Insight. 2021 May 24;6(10):e138027. doi: 10.1172/jci.insight.138027 (PMC8262338; doi:10.1172/jci.insight.138027)
Supplement: Supplemental data [file jciinsight-6-138027-s059.pdf]

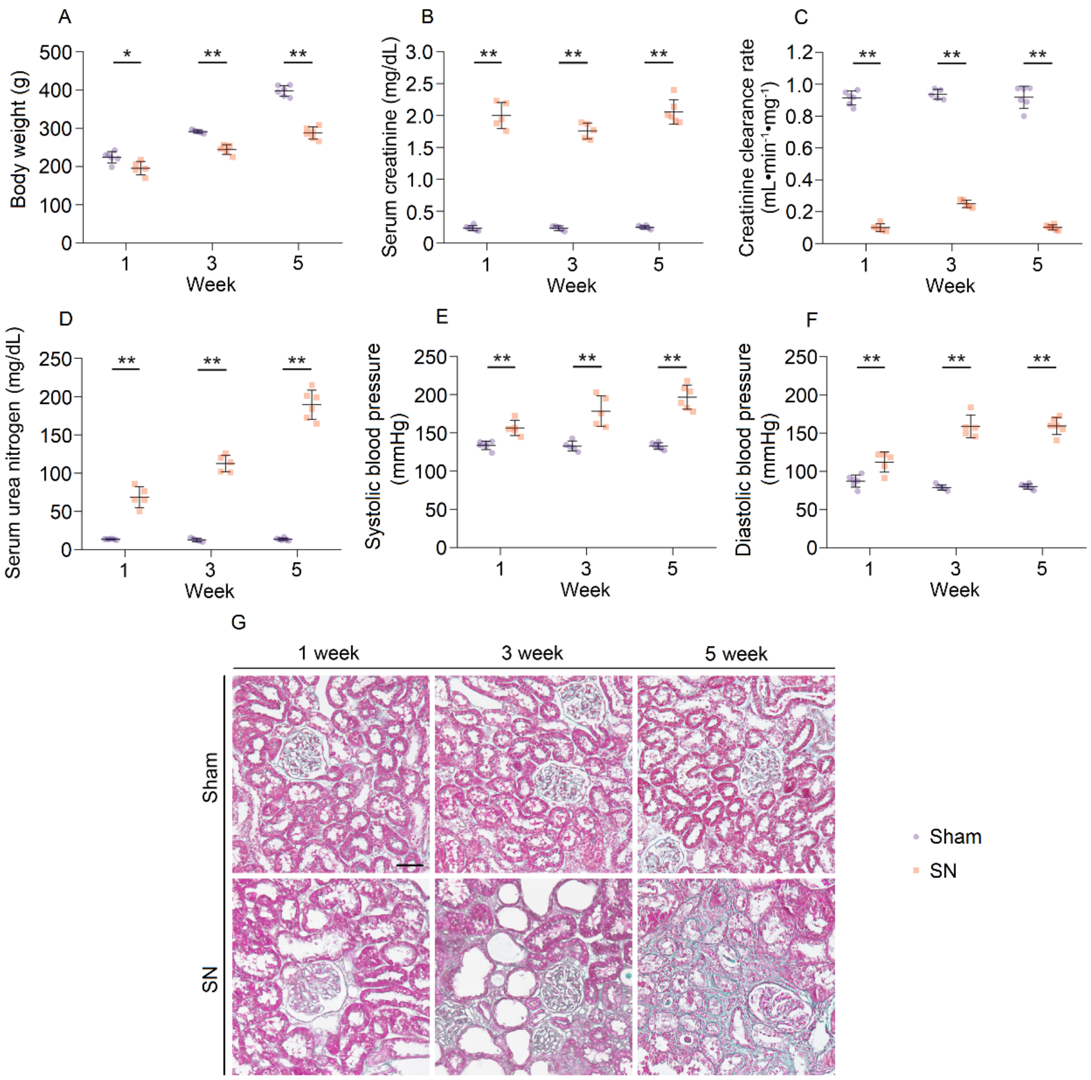

Supplemental Figure 1. SN rats gradually evolve into CKD. (A) Nephrectomized rats exhibit a delayed body growth. *P<0.05 and **P<0.01 compared with values for the sham indicated by the dashed line, by Two-tailed, unpaired Student's *t* test. Data are shown as mean ± SD. n = 5 or 6 rats per group. **(B)** Nephrectomy causes obvious increase in serum creatinine. **P<0.01 compared with values for the sham indicated by the dashed line, by Two-tailed, unpaired Student's *t* test. Data are shown as mean ± SD. n = 5 or 6 rats per group. **(C)** Creatinine clearance rate indicates a marked decrease in SN rats. **P<0.01 compared with values for the sham indicated by the dashed line, by Two-tailed, unpaired Student's *t* test. Data are shown as mean ± SD. n = 5 or 6 rats per

group. **(D)** Nephrectomy causes obvious increase in serum creatinine urea nitrogen. **P<0.01 compared with values for the sham indicated by the dashed line, by Two-tailed, unpaired Student's *t* test. Data are shown as mean ± SD. n = 5 or 6 rats per group. **(E~F)** Blood pressure shows a gradually increasing trend in nephrectomized rats. **P<0.01 compared with values for the sham indicated by the dashed line, by Two-tailed, unpaired Student's *t* test. Data are shown as mean ± SD. n = 5 or 6 rats per group. **(G)** Representative histology of remnant kidney reveals a trend of gradual tubular dilation and interstitial fibrosis in nephrectomized rats. (original magnification, ×100; scale bar: 100 nm)

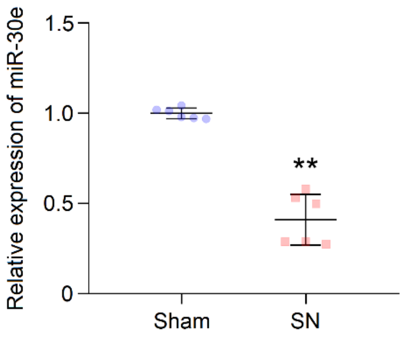

Supplemental Figure 2. SN rats demonstrate cardiac miR-30e suppression at 5 weeks after nephrectomy. Expression levels are normalized by *U6*. **P<0.01 compared with values for the sham, by Two-tailed, unpaired Student's *t* test. Data are shown as mean ± SD. n = 6 rats per group.

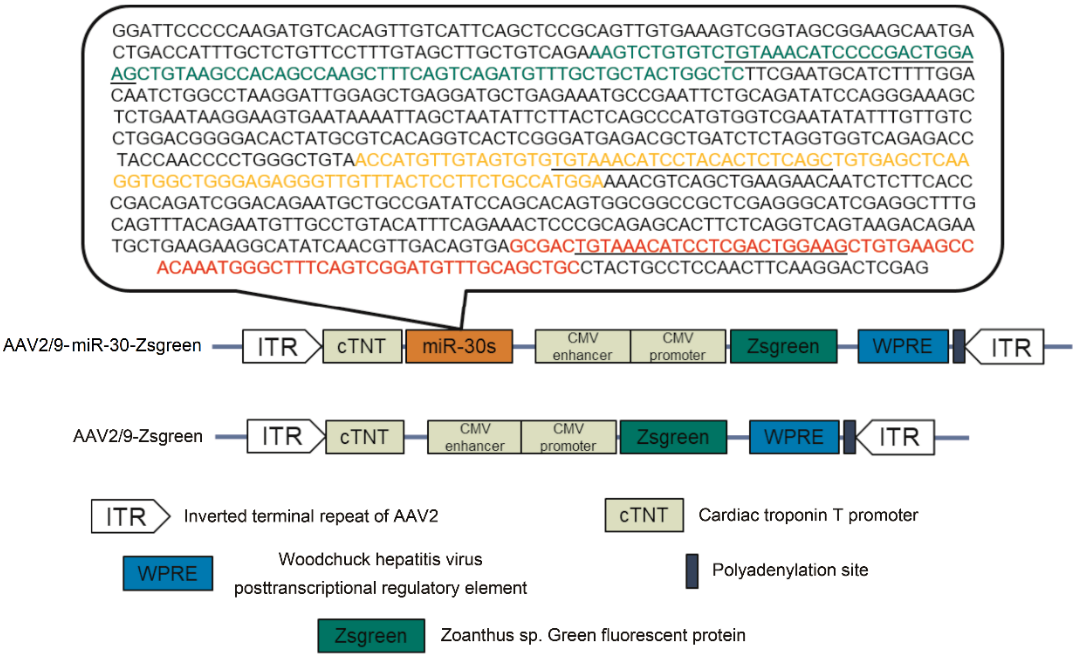

Supplemental Figure 3. The genome structure of AAV2/9. Alphabets with green font, yellow font and red font represent pre-miR-30d, pre-miR-30c and pre-miR-30a, respectively. Underlined letters among them mean mature miR-30d, miR-30c and miR-30a.

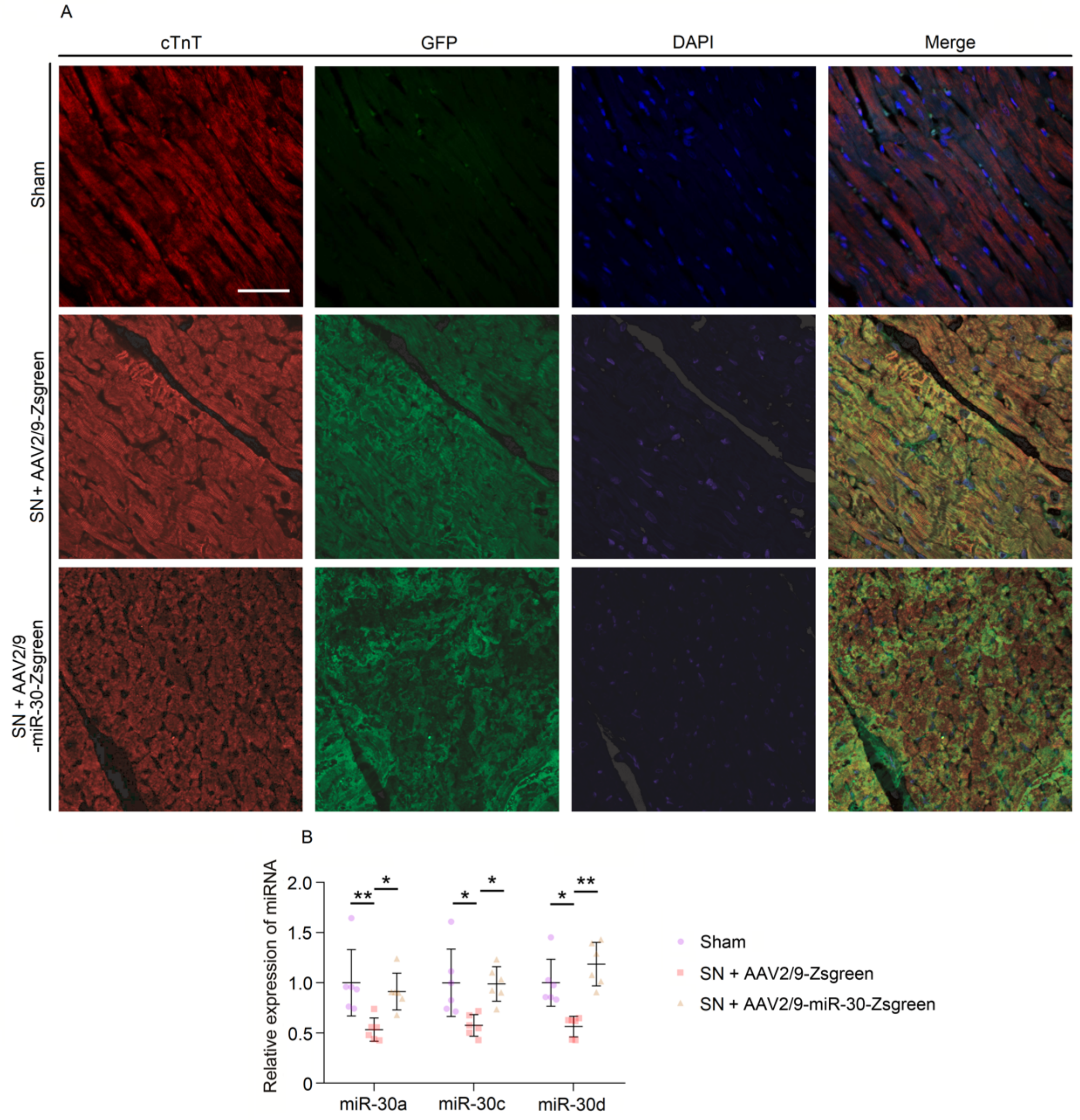

Supplemental Figure 4. AAV2/9-transfected cardiac tissues successfully express miR-30. (A) Zsgreen expresses in cardiomyocytes of rats received AAV2/9 injection (original magnification, ×400; scale bar: 50 nm). **(B)** AAV2/9-miR-30-Zsgreen injection successfully rescues the downregulation of miR-30 in cardiomyocytes of SN rats. Expression levels are normalized by *U6*. *P<0.05 and **P<0.01 compared with values indicated by the dashed line, by One-way ANOVA test. Tukey's multiple comparisons test was used for multiple comparison. Data are shown as mean ± SD. n = 6 rats per group.

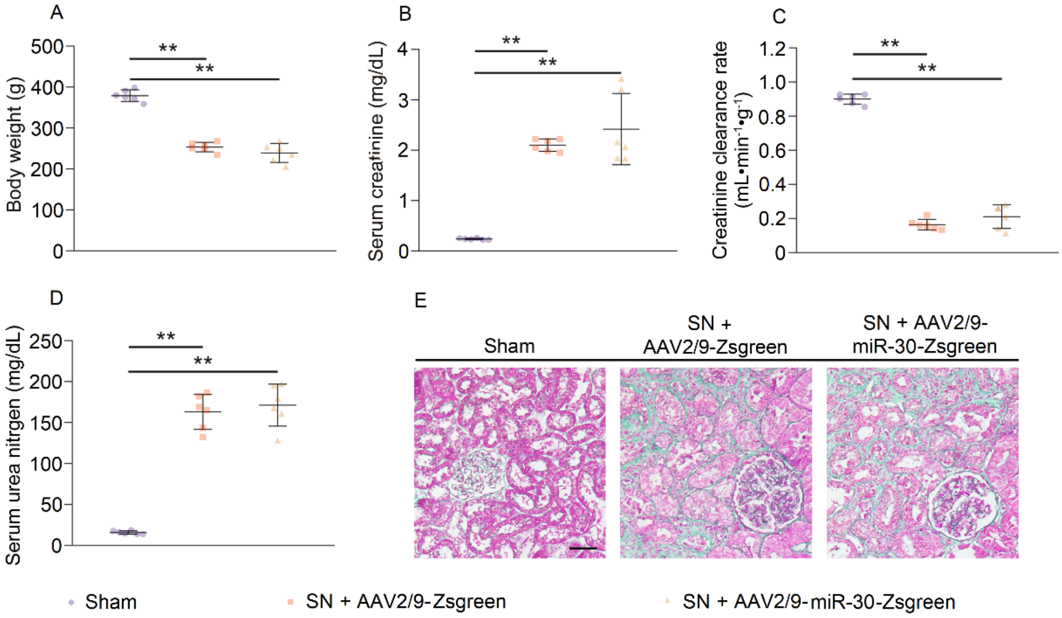

Supplemental Figure 5. AAV2/9 injection does not alternate CKD progression. **(A~D)** AAV2/9 injection does not affect body weight reduction, serum creatinine increment, creatinine clearance rate reduction and serum urea nitrogen increment in SN rats. **P<0.01 compared with values indicated by the dashed line, by One-way ANOVA test. Tukey’s multiple comparisons test was used for multiple comparison. Data are shown as mean ± SD. n = 6 rats per group. **(E)** No reduction in renal fibrosis or morphology changed in nephrectomized groups after AAV2/9 injection (original magnification, ×100; scale bar: 100 nm).

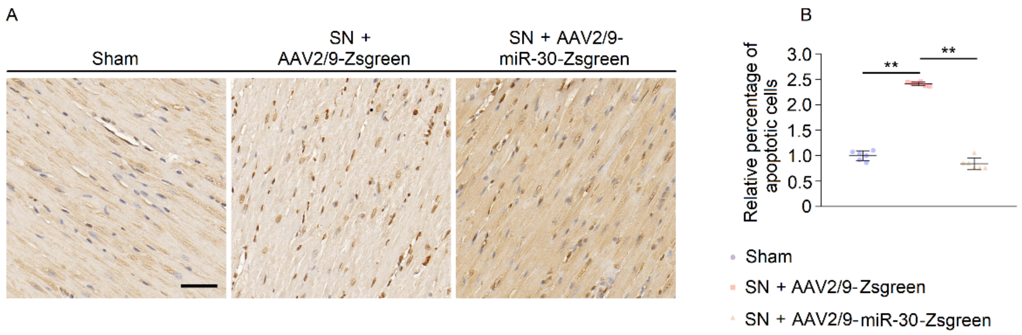

Supplemental Figure 6. Cardiac miR-30 rescue mitigates cardiomyocyte apoptosis in CKD. (A) Representative images of TUNNEL staining (original magnification, ×400; scale bar: 50 nm). **(B)** Quantitative results for TUNNEL staining. **P<0.01 compared with values indicated by the dashed line, by One-way ANOVA test. Tukey's multiple comparisons test was used for multiple comparison. Data are shown as mean ± SD. n = 6 rats per group.

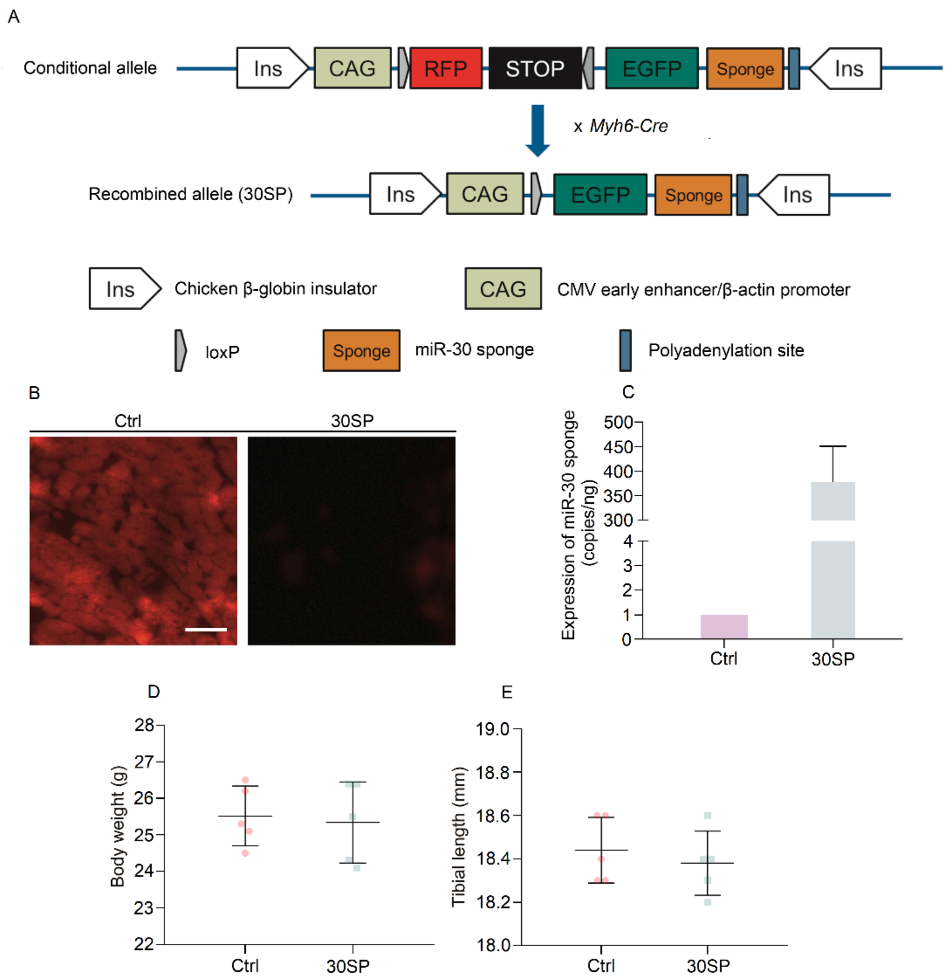

Supplemental Figure 7. 30SP mice express miR-30 sponge in myocardial tissues successfully. (A) The structure of the conditional miR-30 sponge transgene and the product of recombination induced by *Myh6-Cre*, which leads to miR-30 sponge expression in cardiomyocytes specifically. **(B)** Crossing the miR-30 sponge transgenic mice with *Myh6-Cre* transgenic mice results in the loss of RFP expression in cardiomyocytes (original magnification, ×100; scale bar: 100 nm). **(C)** miR-30 sponge expression in the myocardial tissues from 30SP mice (mean ± SD; n = 5 mice per group). **(D~E)** No significant differences in body weight and tibial length between 30SP mice and ctrl mice. Compared with values for the ctrl, by Two-tailed, unpaired Student's *t* test. Data are shown as mean ± SD. n = 5 mice per group.

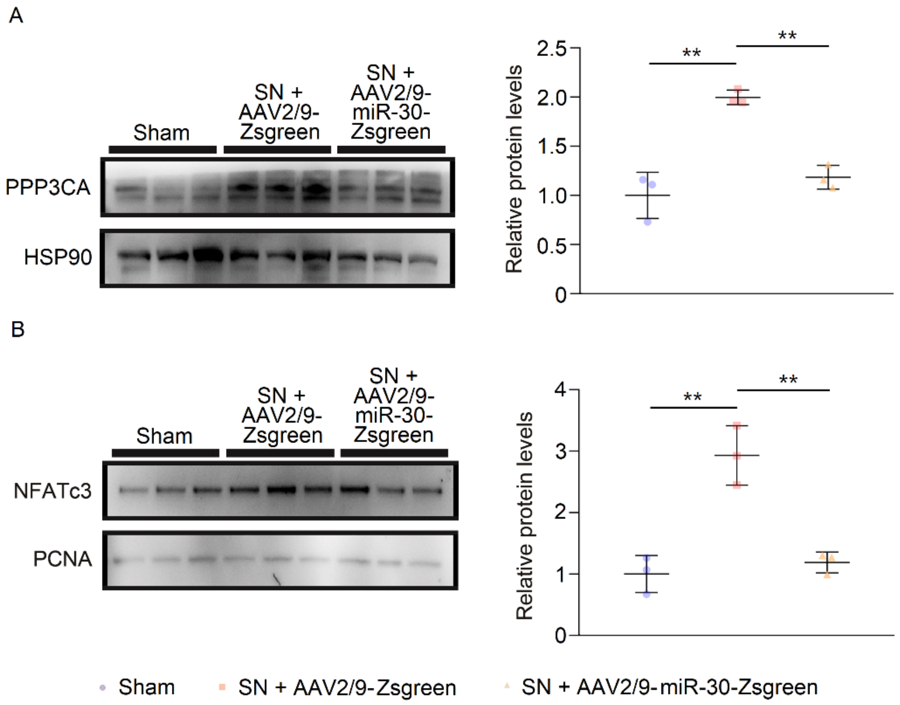

Supplemental Figure 8. Cardiac miR-30 rescue inactivates calcineurin signaling in CKD. (A) miR-30 reduces the upregulation of PPP3CA in myocardium from CKD rats. Protein levels are normalized by HSP90. **P<0.01 compared with values indicated by the dashed line, by One-way ANOVA test. Tukey's multiple comparisons test was used for multiple comparison. Data are shown as mean ± SD. n = 3 independent experiments per group. **(B)** miR-30 inhibits the nuclear translocation of NFATc3 in myocardium from CKD rats. Protein levels are normalized by PCNA. **P<0.01 compared with values indicated by the dashed line, by One-way ANOVA test. Tukey's multiple comparisons test was used for multiple comparison. Data are shown as mean ± SD. n = 3 independent experiments per group.

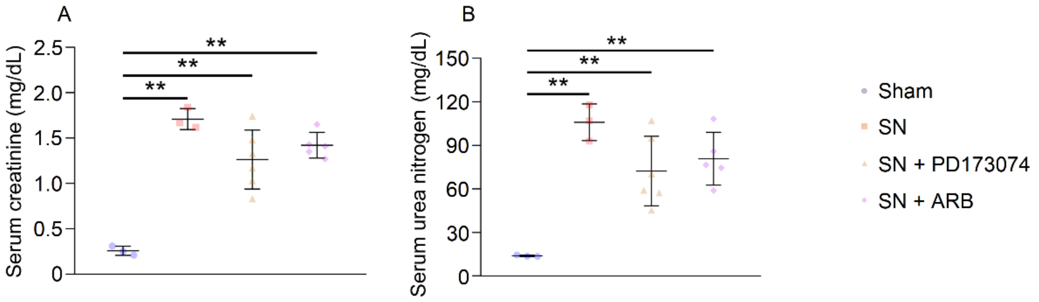

Supplemental Figure 9. PD173074 and ARB administration do not affect kidney impairment in CKD. (A~B) PD173074 and ARB do not significantly affect serum creatinine and urea nitrogen in SN rats. **P<0.01 compared with values indicated by the dashed line, by One-way ANOVA test. Tukey's multiple comparisons test was used for multiple comparison. Data are shown as mean ± SD. n = 3 to 6 rats per group.

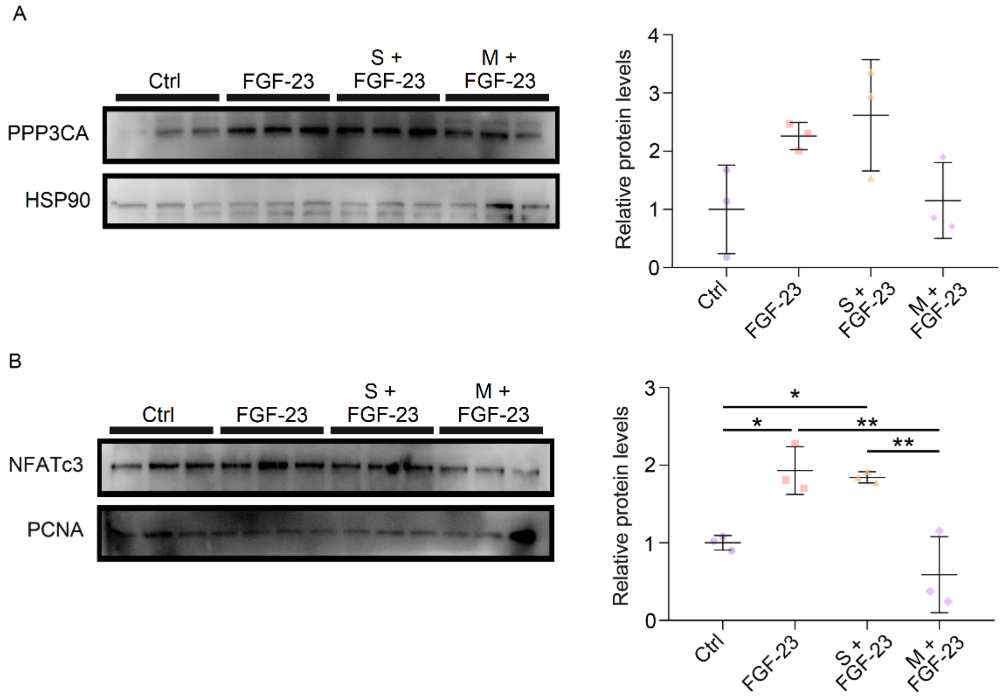

Supplemental Figure 10. MiR-30 mimics inactivates calcineurin signaling in FGF-23-treated NRVMs. (A) miR-30 has no significant effect on PPP3CA expression in FGF-23-treated NRVMs. Protein levels are normalized by HSP90. Compared with values by One-way ANOVA test. Tukey's multiple comparisons test was used for multiple comparison. Data are shown as mean ± SD. n = 3 independent experiments per group. **(B)** miR-30 inhibits the nuclear translocation of NFATc3 i in FGF-23-treated NRVMs. Protein levels are normalized by PCNA. *P<0.05 and **P<0.01 compared with values by One-way ANOVA test. Tukey's multiple comparisons test was used for multiple comparison. Data are shown as mean ± SD. n = 3 independent experiments per group.

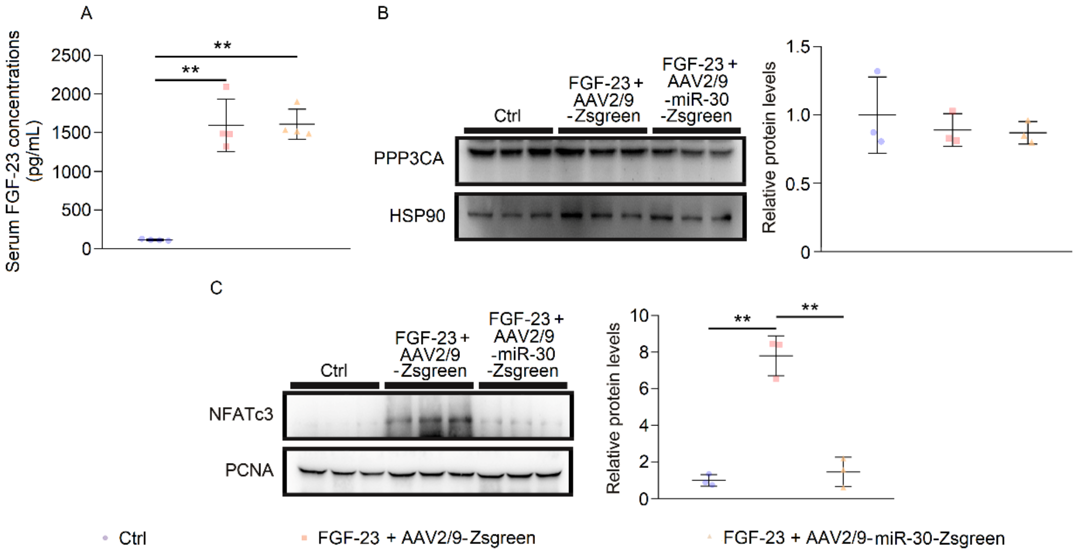

Supplemental Figure 11. Cardiac miR-30 rescue inactivates Calcineurin signaling in FGF-23-treated mice. (A) AAV2/9 injection does not affect circulating levels of FGF-23 in FGF-23-treated mice. **P<0.01 compared with values indicated by the dashed line, by One-way ANOVA test. Tukey's multiple comparisons test was used for multiple comparison. Data are shown as mean ± SD. n = 4 mice per group. **(B)** MiR-30 rescue has no significant effect on cardiac PPP3CA expression in FGF-23-treated mice. Protein levels are normalized by HSP90. Compared with values by One-way ANOVA test. Tukey's multiple comparisons test was used for multiple comparison. Data are shown as mean ± SD. n = 3 independent experiments per group. **(C)** MiR-30 recues inhibits the nuclear translocation of NFATc3 in myocardium from FGF-23-treated mice. Protein levels are normalized by PCNA. **P<0.01 compared with values by One-way ANOVA test. Tukey's multiple comparisons test was used for multiple comparison. Data are shown as mean ± SD. n = 3 independent experiments per group.
